# Supplementary material for: Nr4a1 and Nr4a3 Reporter Mice Are Differentially Sensitive to T Cell Receptor Signal Strength and Duration
Source: Cell Rep. 2020 Nov 3;33(5):108328. doi: 10.1016/j.celrep.2020.108328 (PMC7653457; doi:10.1016/j.celrep.2020.108328)
Supplement: Document S1. Figures S1–S5 [file mmc1.pdf]

Cell Reports, Volume *33*

Supplemental Information

Nr4a1 and Nr4a3 Reporter Mice Are Differentially Sensitive to T Cell Receptor Signal Strength and Duration

Emma Jennings, Thomas A.E. Elliot, Natasha Thawait, Shivani Kanabar, Juan Carlos Yam-Puc, Masahiro Ono, Kai-Michael Toellner, David C. Wraith, Graham Anderson, and David Bending

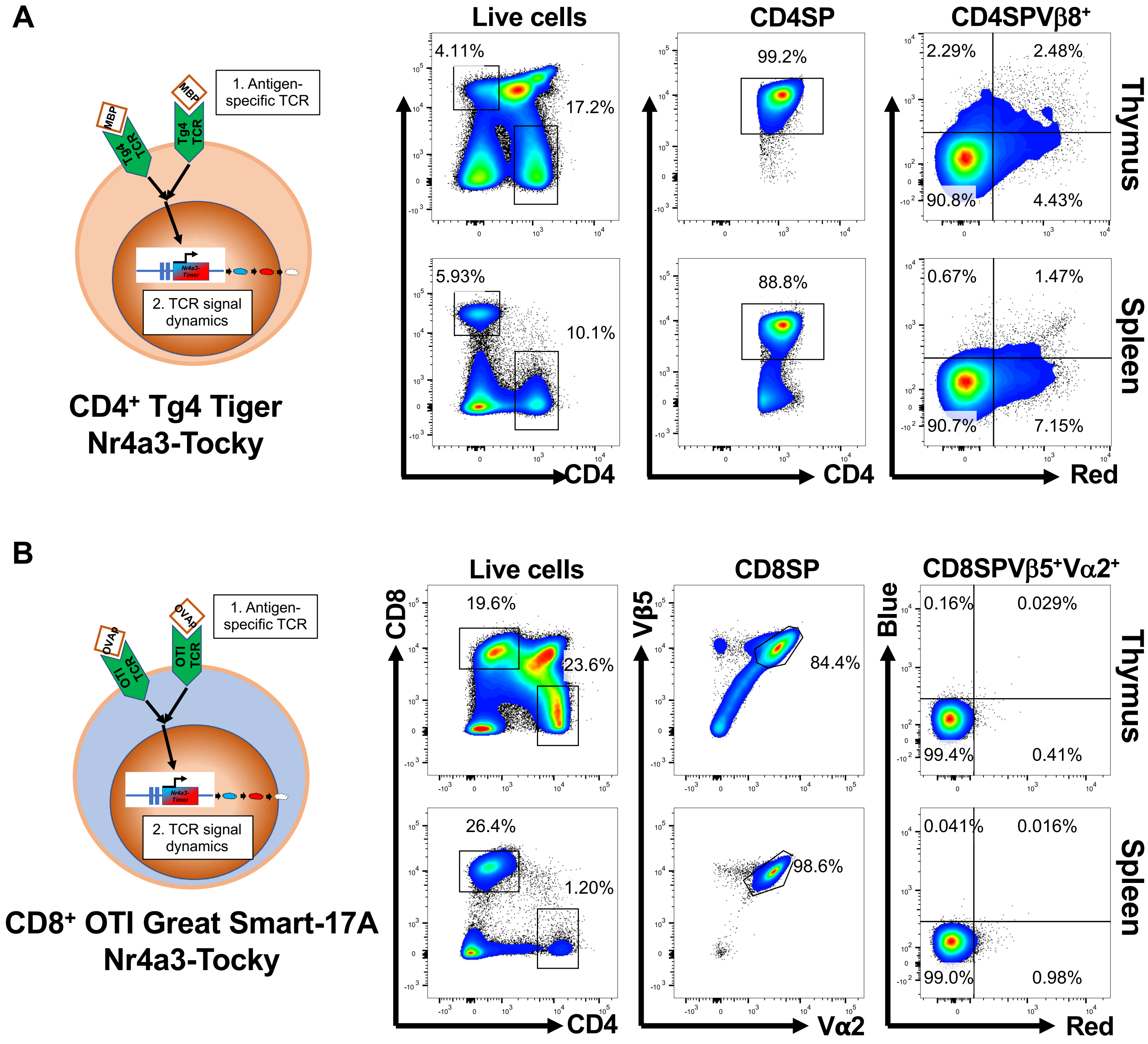

Supplementary Figure 1: Analysis of Tg4 Nr4a3-Tocky and OTI Nr4a3-Tocky mice (related to Figure 1)

(A) Nr4a3-Tocky mice were crossed with Tg4 Tiger mice to generate Tg4 Tiger Nr4a3-Tocky mice, which are specific for myelin basic protein-derived peptides. Thymus (top) and spleen (bottom) were analysed for CD4 vs. CD8 expression within live lymphocytes (left), CD4 vs. TCRVβ8.1/8.2 (middle) gated on CD4SP cells, and Nr4a3-Timer Blue vs Nr4a3-Timer Red in CD4SPVβ8.1/8.2^{+} T cells. **(B)** Nr4a3-Tocky Great Smart-17A mice were bred with OTI TCR transgenic mice, which are specific for ova peptide. Thymus (top) and spleen (bottom) were analysed for CD4 vs CD8 expression within live lymphocytes (left), TCRVβ5.1/5.2 vs TCRVα2 (middle) gated on CD8SP cells, and Nr4a3-Timer Blue vs Nr4a3-Timer Red in CD8SPVβ5.1/5.2^{+}Vα2^{+} T cells.

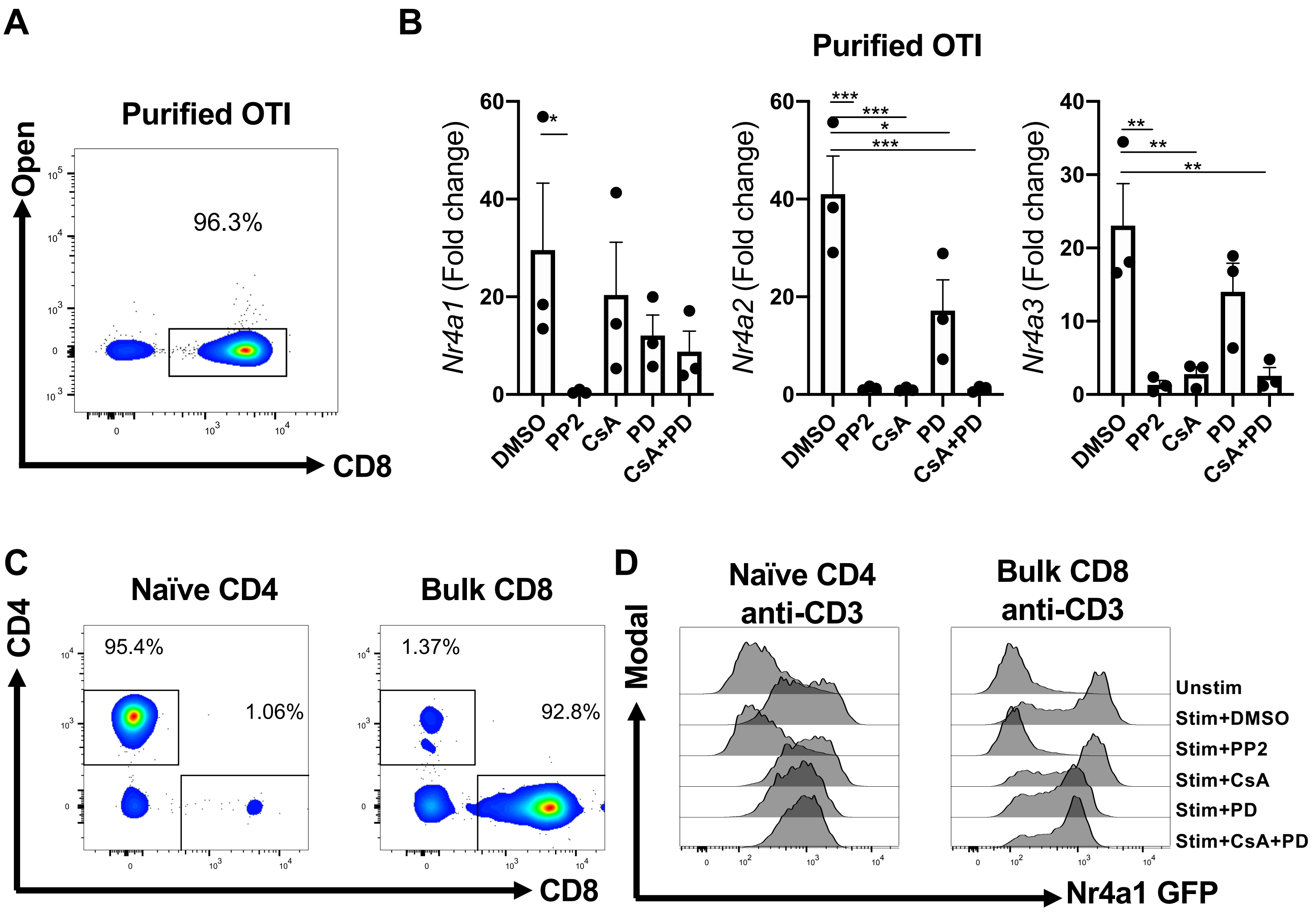

Supplementary Figure 2: Nr4a receptor expression in purified T cell subsets mirrors responses of bulk splenocyte populations to TCR signalling pathways (related to Figure 2)

(A) $CD8^+$ T cells from OTI Nr4a3-Tocky mice were isolated by immunomagnetic selection, purity of a representative isolation is shown gated on live lymphocytes. (**B**) OTI Nr4a3-Tocky $CD8^+$ T cells were stimulated with 1μM ova peptide for two hours in the presence of DMSO, 10μM PP2, 1μM Cyclosporin A (CsA), 5μM PD0325901 (PD) MEK inhibitor or 1μM Cyclosporin A (CsA)+5μM PD0325901 (PD) MEK inhibitor before RNA extraction. Transcript levels of *Nr4a1, Nr4a2* and *Nr4a3* were measured by qPCR and fold change in expression calculated based on unstimulated controls. n=3, bars represent mean±SEM. Statistical analysis by one-way Anova with Tukey's multiple comparisons test. **(C)** Naïve CD4 (left) or bulk CD8 (right) T cells were isolated by immunomagnetic selection from spleens of Nur77-GFP Nr4a3-Tocky mice, representative purity plots based on CD4 and CD8 expression are shown, hgated on live lymphocyte populations. (**D**) T cell populations were stimulated with 5μg/ml soluble anti-CD3 for six hours in the presence of 0.1% DMSO, 10μM PP2, 1μM Cyclosporin A (CsA), 5μM PD0325901 (PD) MEK inhibitor or 1μM Cyclosporin A (CsA)+5μM PD0325901 (PD) MEK inhibitor. Nr4a1-GFP levels in $CD4^+$ (left) or $CD8^+$ right T cells was then evaluated by flow cytometry. Data in (D) are representative of two independent experiments.

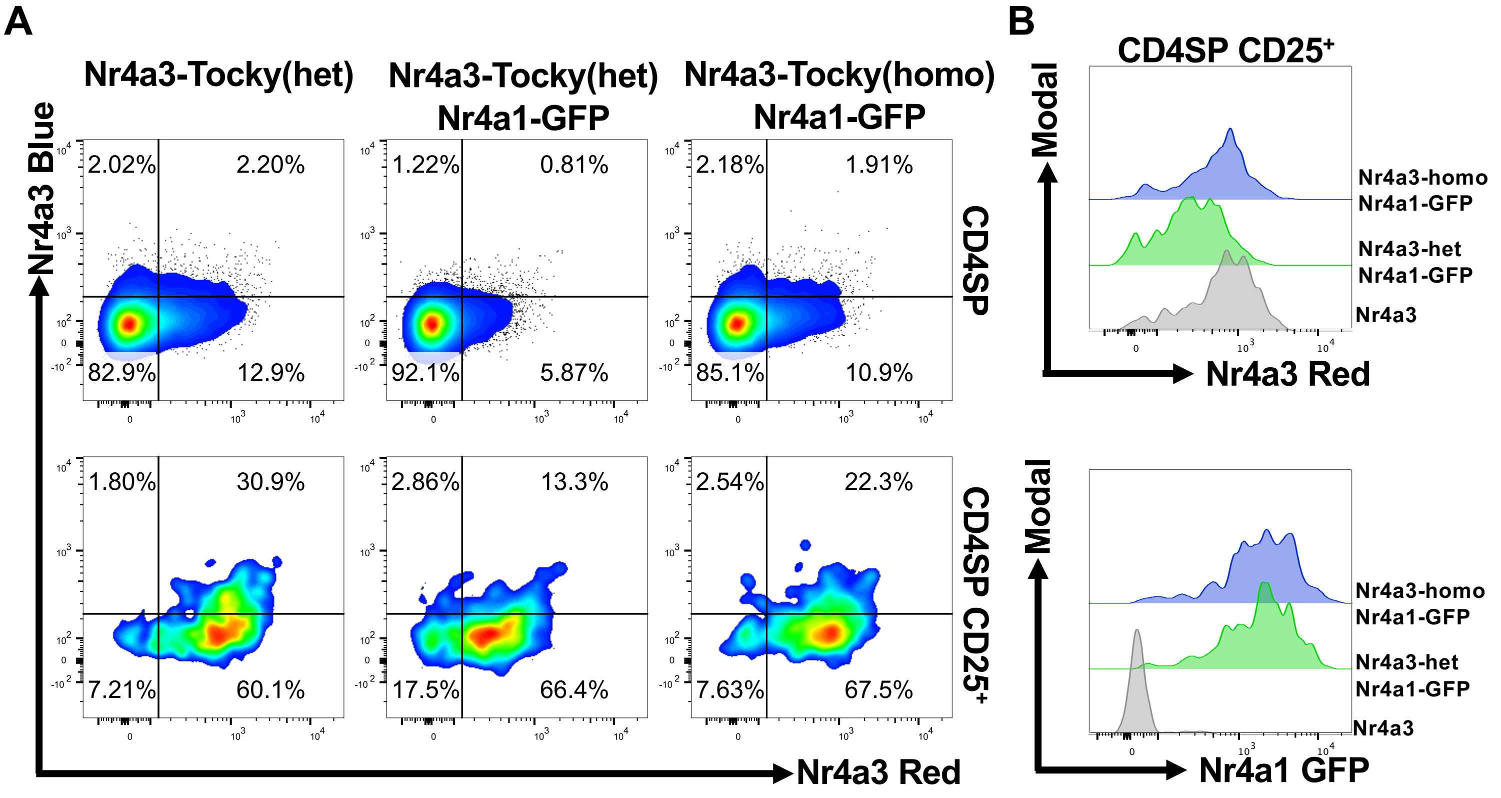

Supplementary Figure 3: Partial quenching of Nr4a3-Tocky expression when crossed with Nr4a1-GFP line (related to Figure 3)

(**A**) Nr4a1-GFP Nr4a3-Tocky mice were generated with either heterozygous or homozygous BAC genetic status. CD4SP and CD4SP CD25$^+$ T cells within the thymus were compared for Nr4a3-Blue vs. Nr4a3-Red expression. (**B**) Histogram overlays showing Nr4a3-Red (top) or Nr4a1-GFP (bottom) in Nr4a3-heterozygous or Nr4a3-homozygous lines carrying the Nr4a1-GFP transgene.

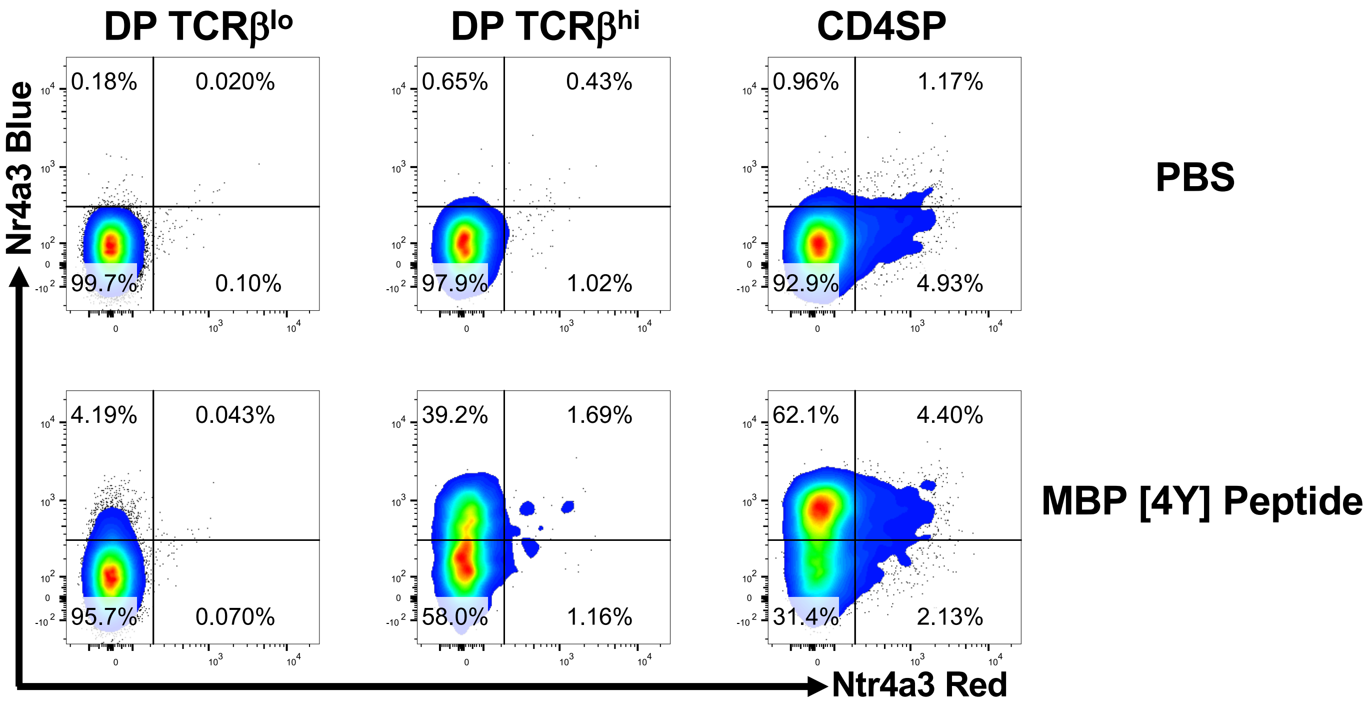

Supplementary Figure 4: Peptide administration activates Nr4a3 expression in developing thymocytes (related to Figure 3)

Tg4 Nr4a3-Tocky mice were immunised with 80µg of MBP [4Y] peptide or vehicle control (PBS) and thymus removed 4hrs later and TCRβ^{lo} and TCRβ^{hi} DP and CD4SP subsets analysed for the expression of Nr4a3-Timer Blue vs Timer Red by flow cytometry.

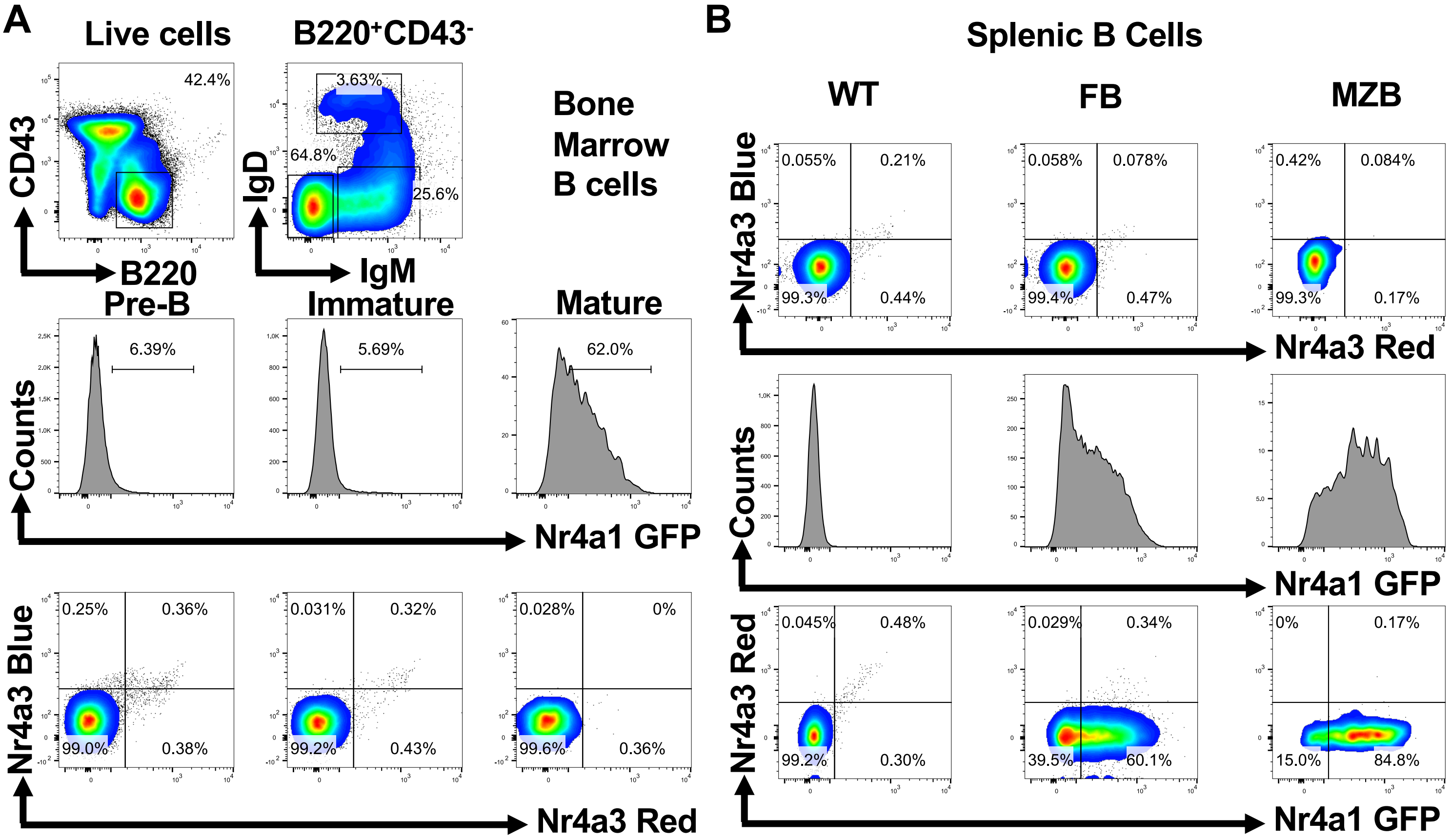

Supplementary Figure 5: Nr4a1 and Nr4a3 expression patterns during B cell development (related to Figure 3)

(A) Bone marrow from Nr4a1-GFP Nr4a3-Tocky mice was analysed for expression of Nr4a1-GFP and Nr4a3-Timer blue and Nr4a3-Timer Red expression within live $B220^{+}CD43^{-}$ B cells. Gating on pre-B cells ($B220^{+}CD43^{-}IgM^{-}IgD^{-}$), immature ($B220^{+}CD43^{-}IgM^{+}IgD^{-}$) and mature B cells ($B220^{+}CD43^{-}IgM^{+}IgD^{+}$) **(B)** Splenic B cell from Nr4a1-GFP Nr4a3-Tocky mice (gated on $B220^{+}CD19^{+)}$ were divided into follicular (FB, $CD21^{+}CD23^{-}$) and marginal zone (MZB, $CD21^{+}CD23^{+}$) subsets and analysed for expression of Nr4a1-GFP and Nr4a3-Timer Blue and Nr4a3-Timer Red expression by flow cytometry. Data are representative of two independent experiments.
