# Supplementary material for: Nr4a1 and Nr4a3 Reporter Mice Are Differentially Sensitive to T Cell Receptor Signal Strength and Duration
Source: Cell Rep. 2020 Nov 3;33(5):108328. doi: 10.1016/j.celrep.2020.108328 (PMC7653457; doi:10.1016/j.celrep.2020.108328)
Supplement: Document S2. Article plus Supplemental Information [file mmc2.pdf]

Cell Reports

Report

# Nr4a1 and Nr4a3 Reporter Mice Are Differentially Sensitive to T Cell Receptor Signal Strength and Duration

## Graphical Abstract

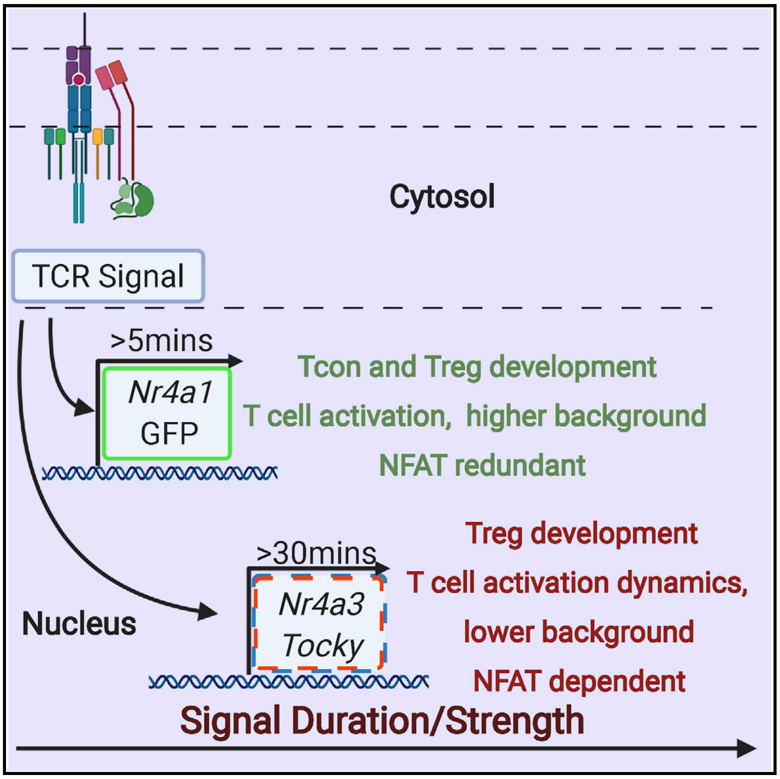


## Highlights

- *Nr4a1* and *Nr4a3* show differential dependency on the calcineurin/NFAT pathway
- Nr4a1-GFP is expressed in developing Tcon and Treg within the thymus
- Nr4a3-Timer expression is largely restricted to thymic and peripheral CD25$^+$ Treg
- Nr4a3-Timer requires a stronger and/or longer TCR signal for its expression


## Authors

Emma Jennings, Thomas A.E. Elliot, Natasha Thawait, ..., David C. Wraith, Graham Anderson, David Bending

## Correspondence

d.a.bending@bham.ac.uk



## In Brief

*Nr4a* reporter mice are useful tools for studying TCR signaling. Jennings et al. show that *Nr4a3*, but not *Nr4a1*, is NFAT pathway dependent, resulting in restricted Nr4a3-Timer expression during T cell development. Nr4a3-Timer requires stronger and/or longer TCR signals than Nr4a1-GFP, allowing an investigation of different TCR signaling grades *in vivo*.


Jennings et al., 2020, Cell Reports *33*, 108328
November 3, 2020 © 2020 The Author(s).
https://doi.org/10.1016/j.celrep.2020.108328

CellPress

Cell Reports

CellPress
OPEN ACCESS

Report

# Nr4a1 and Nr4a3 Reporter Mice Are Differentially Sensitive to T Cell Receptor Signal Strength and Duration

Emma Jennings,[1,3,4] Thomas A.E. Elliot,[1,3] Natasha Thawait,[1] Shivani Kanabar,[1] Juan Carlos Yam-Puc,[1] Masahiro Ono,[2] Kai-Michael Toellner,[1] David C. Wraith,[1] Graham Anderson,[1] and David Bending[1,5,6,*]
[1]Institute of Immunology and Immunotherapy, College of Medical and Dental Sciences, University of Birmingham, Birmingham, B15 2TT, UK
[2]Department of Life Sciences, Imperial College London, London, SW7 2AZ, UK
[3]These authors contributed equally
[4]Present address: Leeds Institute for Medical Research, Faculty of Medicine and Health, University of Leeds, St James' University Hospital, Leeds, LS9 7TF, UK
[5]Twitter: @BendingLab
[6]Lead Contact
*Correspondence: d.a.bending@bham.ac.uk
https://doi.org/10.1016/j.celrep.2020.108328

## SUMMARY

*Nr4a* receptors are activated by T cell receptor (TCR) signaling and play key roles in T cell differentiation. Which TCR signaling pathways regulate Nr4a receptors and their sensitivities to TCR signal strength and duration remains unclear. Using Nr4a1/Nur77-GFP and *Nr4a3*-Timer of cell kinetics and activity (Tocky) mice, we elucidate the signaling pathways governing Nr4a receptor expression. We reveal that *Nr4a1*–*Nr4a3* are Src family kinase dependent. Moreover, *Nr4a2* and *Nr4a3* are attenuated by calcineurin inhibitors and bind nuclear factor of activated T cells 1 (NFAT1), highlighting a necessary and sufficient role for NFAT1 in the control of *Nr4a2* and *Nr4a3,* but redundancy for *Nr4a1*. *Nr4a1*-GFP is activated by tonic and cognate signals during T cell development, whereas *Nr4a3*-Tocky requires cognate peptide:major histocompatibility complex (MHC) interactions for expression. Compared to *Nr4a3*-Tocky, *Nr4a1*-GFP is approximately 2- to 3-fold more sensitive to TCR signaling and is detectable by shorter periods of TCR signaling. These findings suggest that TCR signal duration may be an underappreciated aspect influencing the developmental fate of T cells *in vivo*.

## INTRODUCTION

The Nr4a family of orphan nuclear receptors consists of three members: Nr4a1 (Nur77), Nr4a2 (Nurr1), and Nr4a3 (Nor1). Nr4a receptors are ligand independent and their structure is set to a constitutively active form (Wang et al., 2003). Nr4a1 and Nr4a3 are rapidly upregulated in T cells (Ashouri and Weiss, 2017; Bending et al., 2018b; Moran et al., 2011; Zikherman et al., 2012) and thymocytes (Cheng et al., 1997), following T cell receptor (TCR) signaling, and are a more specific marker of T cell activation than CD69, which is upregulated by non-TCR stimuli (Moran et al., 2011). Expression of *Nr4a1* (Moran et al., 2011) and *Nr4a3* (Bending et al., 2018b) in CD4$^+$ T cells is lost in major histocompatibility complex (MHC) class II knockout mice, highlighting the key role of TCR signaling in regulating Nr4a receptor expression.

Nr4a receptors play important roles in T cell biology. The Nr4a1 protein drives apoptosis (Liu et al., 1994) by associating with Bcl-2 in the mitochondria (Thompson and Winoto, 2008) and can modulate regulatory T cell (Treg) differentiation and clonal deletion (Fassett et al., 2012). Nr4a2 binds *Foxp3* regulatory elements and regulates the differentiation of CD4$^+$ T cells (Sekiya et al., 2011); and persistent *Nr4a3* expression is a hallmark of Treg undergoing differentiation (Bending et al., 2018b). Genetic ablation of all Nr4a receptors abolishes Treg development and can lead to autoimmunity (Sekiya et al., 2013). Recent studies report that nuclear factor of activated T cells (NFAT) (Martinez et al., 2015) and Nr4a receptors (Mognol et al., 2017; Scott-Browne et al., 2016) are linked to CD8$^+$ T cell exhaustion. Indeed, Nr4a family members limit chimeric antigen receptor (CAR) T cell function (Chen et al., 2019) and Nr4a1 drives T cell dysfunction through modulating activator protein one (AP-1) transcription factor activity (Liu et al., 2019). Nr4a receptors also promote CD8$^+$ T cell exhaustion through cooperation with other transcription factors, such as thymocyte-selection-associated high mobility group box protein (TOX) and TOX2 (Seo et al., 2019). In addition, Nr4a1 alters T cell metabolism, acting as a break to dampen inflammation (Liebmann et al., 2018).

Given the central roles of Nr4a members in autoimmunity and cancer, they are an emerging therapeutic target. Pharmacological inhibition of Nr4a receptors enhances antitumor immunity (Hibino et al., 2018). Therefore, understanding the regulation of


This is an open access article under the CC BY license (http://creativecommons.org/licenses/by/4.0/).

Nr4a receptors is of both fundamental and therapeutic interest. *Nr4a* members are paralogs, but the signaling pathways that regulate their expression are ill defined; however, NFAT1 is linked to their function (Martinez et al., 2015; Mognol et al., 2017; Scott-Browne et al., 2016). Furthermore, how TCR signal strength and duration modulate their expression is unknown. Here, we use *Nr4a3*-Timer of cell kinetics and activity (Tocky) (Bending et al., 2018a; Bending et al., 2018b) and *Nr4a1*/Nur77-GFP mice (Moran et al., 2011) to determine the pathways regulating *Nr4a1* and *Nr4a3* transcription in T cells. Our findings highlight that the calcineurin (CaN)/NFAT pathway is necessary and sufficient for the induction of *Nr4a2* and *Nr4a3* but redundant for *Nr4a1*. Moreover, although *Nr4a1* is activated in response to basal TCR signaling during lymphocyte development, *Nr4a3* activation in mature T cells requires a longer TCR signaling period that is achieved only through activating TCR signaling.

## RESULTS

### *Nr4a* Receptors Bind NFAT1 but Only *Nr4a2* and *Nr4a3* Are Calcineurin Pathway Dependent

*Nr4a3*-Tocky mice were crossed with Tg4 Tiger mice (Burton et al., 2014), which express an autoreactive CD4$^+$ TCR specific for myelin basic protein (MBP) (Liu et al., 1995). This system allows an analysis of TCR signaling in response to self-agonist peptides (Figure S1A). In addition, to study CD8$^+$ T cells, *Nr4a3*-Tocky mice were bred to OTI mice (Hogquist et al., 1994; Figure S1B). Stimulation of splenocytes with MBP Ac1-9 [4Y] peptide (Tg4) or ova peptide (OTI) triggered *Nr4a3* expression, resulting in Blue fluorescence, before time-dependent maturation to a Blue$^+$Red$^+$ state (Figures 1A and 1B). Expression of *Nr4a3* was dependent on the Src family kinases because incubation of Tg4 *Nr4a3*-Tocky T cells with the inhibitor PP2 (Hanke et al., 1996) abolished *Nr4a3* and CD69 (Figures 1C and 1D).

TCR signaling results in the activation of many signaling intermediaries but converge on the activation of three key transcription factors: NFAT, AP1, and nuclear factor κB (NF-κB) (Brownlie and Zamoyska, 2013). Given that Nr4a receptors have been linked to NFAT activation (Scott-Browne et al., 2016), we investigated the sensitivity of *Nr4a3* to NFAT pathway inhibitors. We used two distinct inhibitors of Calcineurin (CaN), a key enzyme in TCR signaling that dephosphorylates NFAT in response to $Ca^{2+}$ signaling (Hogan et al., 2003). Incubation with cyclosporin A (CsA; a fungus-derived product that forms a complex with cyclophilin to block the phosphatase activity of CaN; Liu et al., 1991; Matsuda and Koyasu, 2000) or FK506 (a macrolide CaN inhibitor; Thomson et al., 1995) attenuated TCR-stimulation-induced Nr4a3-Timer Blue expression in CD4$^+$ Tg4 T cells (Figures 1E–1G). Highly similar findings were mirrored for CD8$^+$ T cells (Figures 1H–1J). These data suggested that *Nr4a3*-Tocky is an NFAT responsive distal TCR signaling reporter.

Three isoforms of NFAT are expressed in T cells (NFAT1, NFAT2, and NFAT4; Macian, 2005). To ascertain NFAT binding, we identified a previously published chromatin immunoprecipitation sequencing (ChIP-seq) dataset (Gene Expression Omnibus (GEO) GEO: GSE64409; Martinez et al., 2015) for the binding of NFAT1 to *Nr4a* genes. Analysis of NFAT1 binding peaks revealed evidence for NFAT1 binding across all *Nr4a* gene family members (Figure 1K). To test the sensitivity of the other *Nr4a* family members to CaN inhibitors, *Nr4a1*, *Nr4a2*, and *Nr4a3* mRNA levels were quantified in response to TCR stimulation. Although *Nr4a2* and *Nr4a3* showed inhibition of transcriptional upregulation in response to both CsA and FK506, *Nr4a1* was insensitive to both inhibitors, as has been previously reported (Zikherman et al., 2012; Figure 1L). In order to directly compare expression in the same cells, *Nr4a3*-Tocky mice were crossed with *Nr4a1*-GFP mice (Moran et al., 2011) to generate a dual reporter. Splenocytes of *Nr4a1*-GFP/*Nr4a3*-Tocky mice were stimulated for 4 h with soluble anti-CD3 in the presence of a range of CsA or FK506 doses (Figure 1M). Both reporters were highly expressed following stimulation; *Nr4a3*-Tocky expression is markedly reduced at doses in excess of 0.01 μM, whereas Nr4a1-GFP expression remained insensitive even up to doses as high as 10 μM. Therefore, although NFAT1 binds to all *Nr4a* gene family members, NFAT pathway signaling appears necessary only for the expression of *Nr4a2* and *Nr4a3* and is redundant for *Nr4a1*.

### ERK Signaling Is Required for Maximal *Nr4a* Expression, but *Nr4a3* Can Be Activated by NFAT Overexpression Alone

NFAT extensively co-operates with AP-1 (Jain et al., 1993; Peterson et al., 1996), and together they activate important genes in response to TCR stimulation. AP-1 is dependent on mitogen-activated protein kinase (MAPK) activity (Karin, 1995), in particular ERK pathway activation. To test the sensitivity of *Nr4a3* and CD69 to ERK/AP-1 pathway inhibition, purified CD8 T cells from *Nr4a3*-Tocky OTI mice were stimulated with ova peptide in the presence of PP2, CsA, or PD0325901 (PD, an ERK pathway inhibitor; Barrett et al., 2008). As previously shown in bulk splenocytes, *Nr4a3* and CD69 expression are rapidly upregulated following stimulation. TCR-induced *Nr4a3* expression was significantly reduced by NFAT pathway inhibition and partially reduced by ERK pathway inhibition (Figures 2A and 3B). TCR-induced CD69 expression showed partial sensitivity to NFAT and ERK pathway inhibition and complete abolition following inhibition of both pathways (Figures 2A and 2C).

To further analyze the kinetics of *Nr4a* receptor transcription in response to TCR stimulation, a time course analysis was performed on peptide-stimulated Tg4 or OTI T cells (Figures 2D and 2E) in the presence of NFAT or ERK pathway inhibitors. Significant inhibition of all *Nr4a* family member transcription by ERK pathway inhibition occurred at 2 h post-stimulation, but there was no significant effect of CsA on *Nr4a1* transcription. In contrast, CsA resulted in a greater repression of *Nr4a2* and *Nr4a3* transcription than ERK pathway inhibition (Figures 2D and 2E), in keeping with the analysis of Nr4a3-Timer expression by flow cytometry (Figure 2A). OTI T cells largely mirrored Tg4 T cells in the sensitivity of *Nr4a* receptors to NFAT and ERK pathway inhibitors, with all showing partial ERK dependence, but only *Nr4a2* and *Nr4a3* being sensitive to CsA (Figure 2E). Using *Nr4a1*-GFP mice, we confirmed that the BAC *Nr4a1* transgenic reporter also showed partial sensitivity to ERK pathway inhibition but is insensitive to NFAT pathway inhibition (Zikherman et al., 2012; Figure 2F). In order to rule out the possibility that the expression of *Nr4a1* could be modulated by co-stimulation or cytokines derived from antigen-presenting cells (APCs) present in

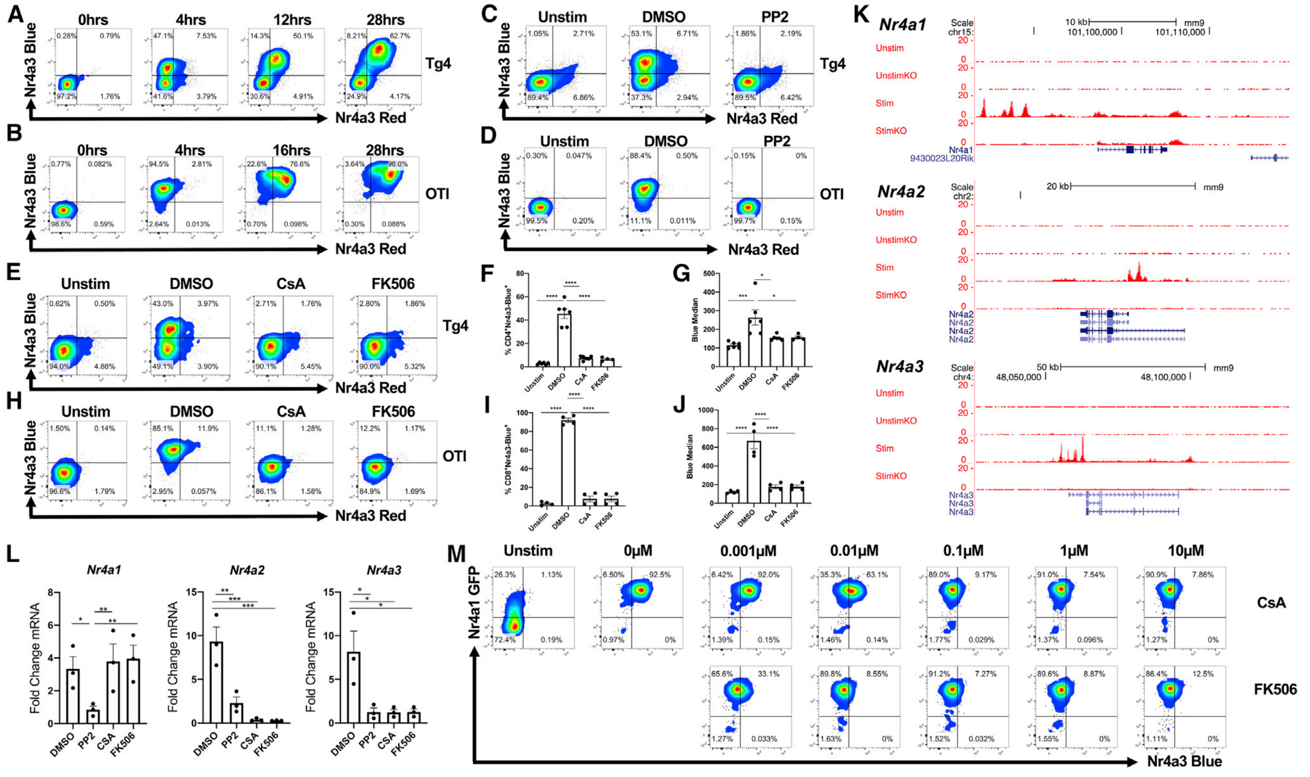


**Figure 1. *Nr4a* Receptors Bind NFAT1, but Only *Nr4a2* and *Nr4a3* Are Calcineurin Pathway Dependent**

(A and B) Splenocytes from Tg4 *Nr4a3*-Tocky mice (A) or OTI *Nr4a3*-Tocky mice (B) were stimulated with 10 μM 4Y-MBP before analysis by flow cytometry for expression of Nr4a3-Timer Blue versus Nr4a3-Timer Red in live T cells.

(C and D) Splenocytes from Tg4 *Nr4a3*-Tocky mice were stimulated with 10 μM 4Y-MBP (C) or OTI *Nr4a3*-Tocky were stimulated with 1 μM ova peptide (D) for 4 h in the presence of DMSO or 10 μM PP2 before analysis by flow cytometry for Nr4a3-Timer Blue versus Nr4a3-Timer Red expression in live CD4$^{+}$ (C) or CD8$^{+}$ (D) T cells.

(E and F) Splenocytes from Tg4 *Nr4a3*-Tocky mice were stimulated with 10 μM 4Y-MBP variant for 4 h in the presence of DMSO, 1 μM cyclosporin A (CsA), or 1 μM FK506 before analysis of Nr4a3-Time Blue versus Nr4a3-Timer Red expression in live CD4$^{+}$ Tg4 T cells by flow cytometry (E). Summary data showing the % Nr4a3-Timer Blue$^{+}$ (F) or median Nr4a3-Timer Blue in live CD4$^{+}$ Tg4 T cells (G); n = 4 (FK506) or n = 6 (unstim [unstimulated control], DMSO, and CsA).

(F and G) Statistical analysis by one-way ANOVA with Tukey's multiple comparisons test. Bars represent mean ± SEM; dots represent individual mice. *p<0.05, ***p<0.001, ****p<0.0001.

(H–J) Splenocytes from OTI *Nr4a3*-Tocky mice were stimulated with 1 μM ova peptide for 4 h in the presence of DMSO, 1 μM CsA, or 1 μM FK506 before analysis of Nr4a3-Timer Blue versus Nr4a3-Timer Red expression in live CD8$^{+}$ OTI T cells by flow cytometry (H). Summary data showing the % Nr4a3-Timer Blue$^{+}$ (I) or median Nr4a3-Timer Blue (J) in live CD8$^{+}$ OTI T cells, n = 4.

(I and J) Statistical analysis by one-way ANOVA with Tukey's multiple comparisons test. Bars represent mean ± SEM; dots represent individual mice. ****p<0.0001.

(K) University of California, Santa Cruz (UCSC) genome browser tracks showing NFAT1 binding peaks in P14$^{+}$ *Tcra*$^{-/-}$ CD8$^{+}$ T cells from GEO: GSE64409. UnstimKO, unstimulated NFAT1 knockout [KO]; Stim, phorbol myristate acetate (PMA) and ionomycin stimulation; StimKO, PMA and ionomycin stimulation in NFAT1KO mice.

(L) Splenocytes from Tg4 *Nr4a3*-Tocky mice were stimulated with 5 μg/ml soluble anti-CD3 for 4 h in the presence of DMSO (–), 10 μM PP2, 1 μM CsA, or 1 μM FK506 before extraction of RNA and analysis of fold change in *Nr4a* receptor transcription; n = 3. Statistical analysis by one-way ANOVA with Tukey's multiple comparisons test. Bars represent mean ± SEM; dots represent individual mice. *p<0.05, **p<0.01, ***p<0.001.

(M) Splenocytes from *Nr4a1*-GFP *Nr4a3*-Tocky mice were stimulated with 5 μg/ml soluble anti-CD3 for 4 h in the presence of CsA or FK506. Live CD8$^{+}$ T cells were analyzed by flow cytometry for expression of Nr4a1-GFP and Nr4a3-Timer Blue. See also Figure S1.

splenocyte cultures, inhibitor studies were verified using purified cells (Figures S2A). Transcript levels of *Nr4a* family member genes were measured in purified *Nr4a3*-Tocky OTI CD8 T cells following stimulation with peptide in the presence of inhibitors (Figure S2B). In addition, naive CD4 and bulk CD8 T cells were purified from *Nr4a1*-GFP mice and stimulated with soluble anti-CD3 before an analysis of *Nr4a1*-GFP expression by flow cytometry (Figures S2C and S2D). In both instances, our previous findings were re-capitulated.

Given that NFAT:AP-1 complexes are critical to T cell activation, we investigated whether NFAT activity alone is sufficient to drive *Nr4a2* and *Nr4a3* expression. *In silico* analysis of a published RNA sequencing (RNA-seq) dataset of T cells expressing constitutively active NFAT1, which was modified to abrogate its binding to AP1 (Martinez et al., 2015; constitutively active R468, I469 and T535 (CA-RIT)-NFAT1), revealed that CA-RIT-NFAT1 resulted in a statistically significant upregulation of *Nr4a3* within CD4 (>3-fold; Figure 2G) and CD8 T cells (>50-fold; Figure 2H)

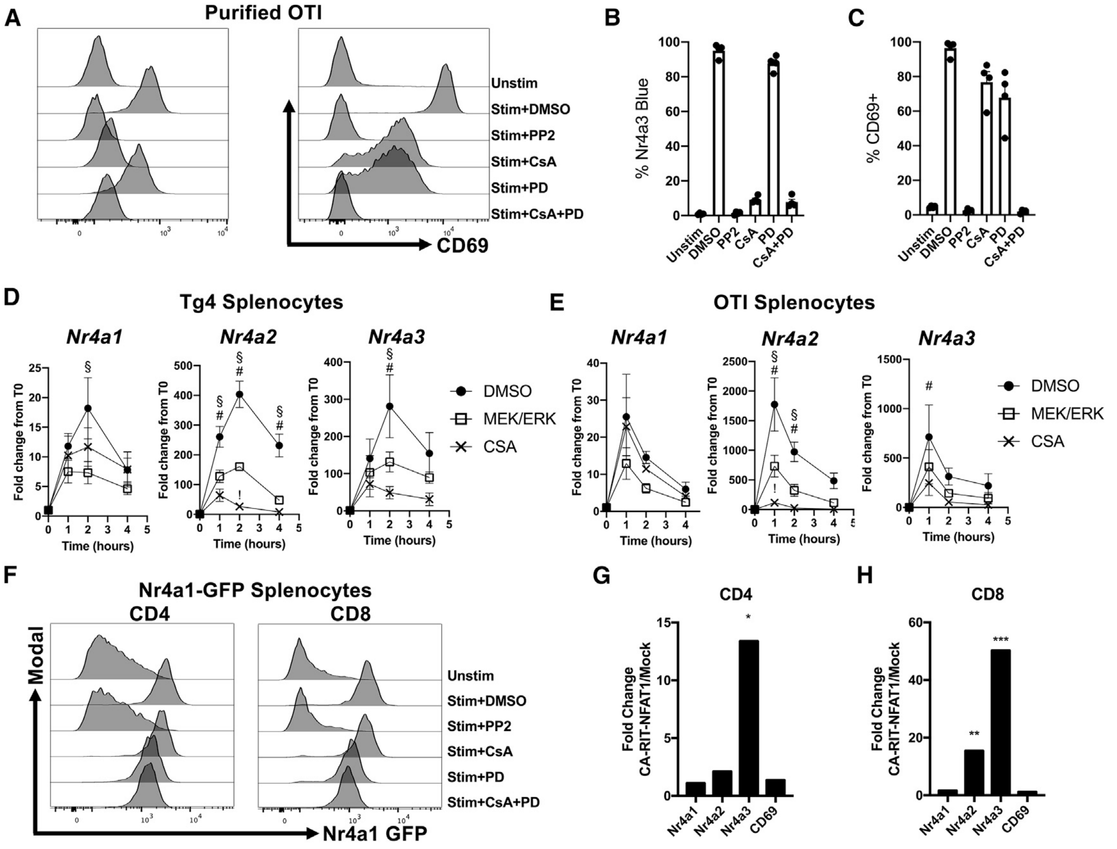


**Figure 2. ERK Signaling Is Required for Maximal *Nr4a* Expression in T Cells, but *Nr4a3* Can Be Activated by NFAT Overexpression Alone**

(A–C) Purified OTI Nr4a3-*Tocky* CD8$^{+}$ T cells were stimulated with 1 μM ova peptide for 4 h in the presence of DMSO, 10 μM PP2, 1 μM CsA, 5 μM PD0325901 (PD), or CsA and PD. Cells were analyzed by flow cytometry for the expression of Nr4a3-Timer Blue and CD69 in live CD8$^{+}$ T cells. Summary data from %Nr4a3-Timer Blue$^{+}$ (B) and %CD69$^{+}$ (C); n = 4. Bars represent mean ± SEM, dots represent individual donor mice. (D–E) Splenocytes from Tg4 *Nr4a3*-Tocky mice were stimulated with 10 μM 4Y-MBP (D) or OTI *Nr4a3*-Tocky mice with 1 μM ova peptide (E) for 4 h in the presence of DMSO, 1 μM CsA, or 5 μM PD; RNA was extracted at 0, 1, 2, and 4 h following culture; and fold change in *Nr4a1–Nr4a3* transcription was measured. DMSO treated, black circles; CsA treated, black crosses; or PD treated, open squares; n = 4 (D); or n = 3 (E). Bars represent mean ± SEM; #, CsA treated significantly reduced ($p < 0.05$) from DMSO; §, PD treated significantly reduced ($p < 0.05$) from DMSO; !, CsA treated significantly reduced ($p < 0.05$) from PD. Statistical analysis by two-way ANOVA with Tukey's multiple comparisons test.

(F) Splenocytes from Nr4a1-GFP mice were cultured for 4 h with 5 μg/ml anti-CD3 or media alone (unstim) in the presence of 0.1% DMSO, 10 μM PP2, 1 μM CsA or 5 μM PD, or 1 μM CsA and 5 μM PD. Live CD4$^{+}$ (left) or CD8$^{+}$ (right) T cells were then analyzed for Nr4a1-GFP expression.

(G and H) Analysis of RNA-seq data from GEO: GSE64409 displaying the fold change in expression of *Nr4a1*, *Nr4a2*, *Nr4a3*, and *Cd69* in CD4$^{+}$ (G) or CD8$^{+}$ (H) T cells transduced with CA-RIT-NFAT1. Adjusted p values based on DESeq analysis; *p = 0.0206, **p = 0.00305, ***p = 0.00016. See also Figure S2.

and *Nr4a2* within CD8s (>15-fold; Figure 2H). Therefore, NFAT1 activity alone is sufficient for the induction of *Nr4a2* and *Nr4a3* expression in CD8 T cells and *Nr4a3* in CD4 T cells, but not *Nr4a1* and *Cd69.*

## *Nr4a1* and *Nr4a3* Expression Patterns during Lymphocyte Development

Given the differential sensitivity of *Nr4a1* and *Nr4a3* to TCR signaling pathways, we interrogated their expression in *Nr4a1*-GFP *Nr4a3*-Tocky mice. A partial quenching of the *Nr4a3*-Tocky reporter was seen when the line was generated as a "heterozygous" bacterial artificial clone (BAC) compared to "homozygous," but similar patterns of expression remained (Figure S3). Thymic T cell development analysis revealed that Nr4a1-GFP was absent within pre-selection immature TCRβ$^{lo}$ double positive (DP) cells, but increased in expression in the TCRβ$^{hi}$ DP fraction, indicative of low-strength TCR signaling events that occur during positive selection, such that all positively selected CD4 single positive (SP) cells expressed Nr4a1-GFP (Figure 3A). Nr4a3 expression was largely absent from the DP and CD4$^{+}$CD25$^{-}$ fraction, showing moderate expression only in Nr4a1-GFP bright cells; however, it was highly expressed within the CD4SP CD25$^{hi}$ fraction, which is enriched with Tregs (Figures 3A and 3D). A comparison of Nr4a1-GFP and Nr4a3-Timer Red revealed how Nr4a3-Timer expression only emerged at the highest levels of GFP, indicating that cells may be receiving different strength/durations of TCR signaling *in vivo*. Importantly, administration of cognate peptide in Tg4 *Nr4a3*-Tocky mice elicited *Nr4a3* expression in both DP and CD4SP thymocytes. This suggests that the lack of *Nr4a3* expression is not due to inaccessibility of the *Nr4a3* BAC reporter locus in thymocytes (Figure S4). In keeping with the thymus, peripheral CD4$^{+}$CD25$^{-}$ and CD8+ T cells

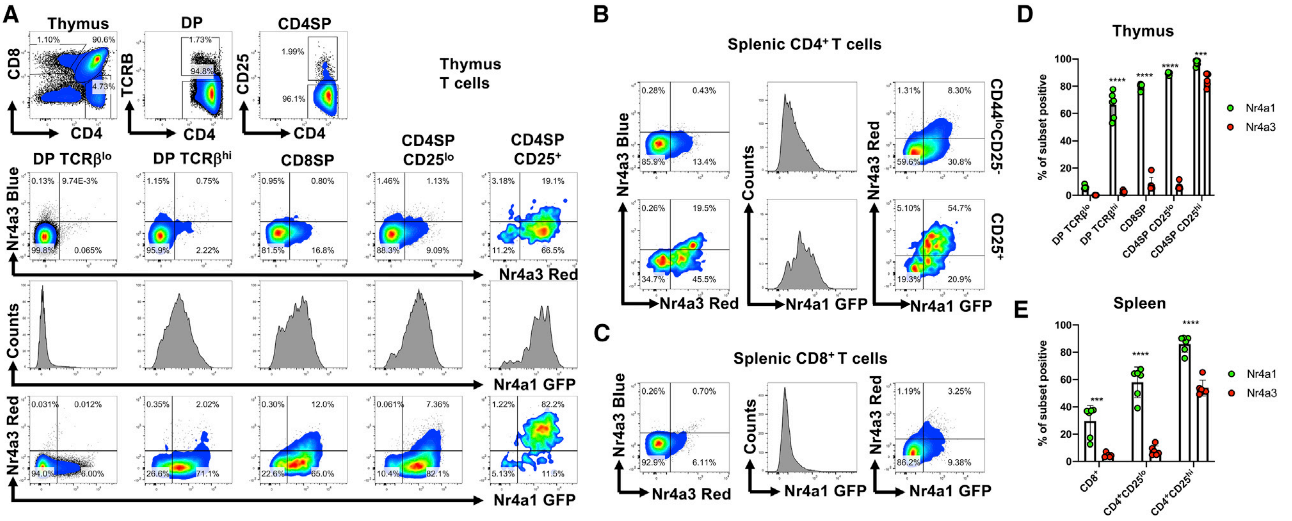


**Figure 3.** ***Nr4a1*** **and** ***Nr4a3*** **Expression Patterns during T Cell Development in the Thymus**

(A) Thymus from *Nr4a1*-GFP *Nr4a3*-Tocky mice was analyzed for expression of Nr4a1-GFP and Nr4a3-Timer Blue and Nr4a3-Timer Red expression within live TCRβ$^{lo}$ and TCRβ$^{hi}$ double positive (DP), CD25$^{-}$ and CD25$^{+}$ CD4SP, and CD8SP subsets by flow cytometry.

(B–E) Splenic CD4$^{+}$CD25$^{-}$, CD4$^{+}$CD25$^{-}$ T cells (B) and CD8$^{+}$ T cells (C) were analyzed for expression of Nr4a1-GFP and Nr4a3-Timer Blue and Nr4a3-Timer Red expression by flow cytometry. Summary data of Nr4a1-GFP and Nr4a3-Timer expression in thymic (D) or splenic (E) T cell subsets. Bars represent mean ± SEM; dots represent individual mice; n = 6. Data are pooled from four independent experiments. Statistical analysis by two-way ANOVA with Tukey's multiple comparisons tests. ***$p < 0.001$, ****$p < 0.0001$. See also Figures S3, S4, and S5.

expressed intermediate Nr4a1-GFP levels and largely lacked Nr4a3 expression. In contrast, CD4$^{+}$CD25$^{+}$ T cells were enriched for Nr4a3$^{+}$ T cells as previously reported (Bending et al., 2018b; Figure 3B). In all peripheral T cell subsets, significant background Nr4a1-GFP expression remained in non-Treg subsets (Figure 3E).

Nr4a1-GFP is also expressed in developing and mature B cells (Moran et al., 2011; Zikherman et al., 2012). Similarly, we found that mature B cells expressed Nr4a1, but not Nr4a3, highlighting that tonic B cell receptor (BCR) signaling can also activate Nr4a1 but not Nr4a3 expression (Figure S5A). Furthermore, in the spleen, marginal zone (MZ) B cells exhibited higher levels of Nr4a1-GFP than follicular (F) B cells, despite continuing to be absent for Nr4a3-Timer expression (Figure S5B).

## *Nr4a1*-GFP Is More Sensitive to TCR Signals and Can Be Activated by Shorter TCR Signaling Bursts than *Nr4a3*-Timer

Based on differential expression of *Nr4a1* and *Nr4a3* in lymphocytes, we hypothesized that *Nr4a1* has a higher sensitivity to TCR signaling. Indeed, it has been reported in CD4 T cells that *Nr4a3* expression has a 3-fold higher half maximal effective concentration ($EC_{50}$) than *Nr4a1* in response to peptide stimulation (Shimizu et al., 2020). In order to verify this finding, we stimulated *Nr4a1*-GFP *Nr4a3*-Tocky splenocytes with a dose titration of soluble anti-CD3 for 4 h (Figure 4A). Dose-response curves of Nr4a1-GFP and Nr4a3-Timer expression showed that the proportion of Nr4a1-GFP$^{+}$ T cells increased at lower doses than Nr4a3-Blue$^{+}$ T cells (Figures 4B and 4C). $EC_{50}$ values were calculated following data normalization, revealing a 2.9-fold difference in $EC_{50}$ between Nr4a1-GFP and Nr4a3-Blue for CD8s (Figure 4C).

This increased sensitivity of *Nr4a1* to TCR signals, however, seemed insufficient to explain the disparity between thymic Nr4a1-GFP and Nr4a3-Timer expression. Given that *Nr4a3* is highly expressed in thymic CD25$^{+}$ Treg cells (Figures 3A and 3D) and our previous findings that thymic Tregs persistently express *Nr4a3* (Bending et al., 2018b), we hypothesized that TCR signal duration may determine Nr4a1-GFP and Nr4a3-Timer expression. We modulated the length of TCR signaling *in vitro* by stimulating *Nr4a1*-GFP *Nr4a3*-Tocky splenocytes with anti-CD3 and then terminating TCR signaling through pharmacological inhibition with PP2 (Figure 4D). Following termination of TCR signaling, T cells remained in culture for 4 h to allow time for translation of the reporter proteins and their accumulation (Figures 4D–4F). The proportion of Nr4a1-GFP-expressing cells increased after 5 min of stimulation, reaching a maximum response at 1 h. The proportion of *Nr4a3*-expressing cells did not increase until 30 min and did not reach maximum until 4 h (Figures 4E and 4F; time to half of maximum [$T_{1/2}$] *Nr4a1* = 12 min, $T_{1/2}$ *Nr4a3* = 70 min). Differential sensitivity of *Nr4a1*-GFP and *Nr4a3*-Tocky to TCR signaling duration suggests that TCR signaling events in the thymus that lead to thymic Treg development must persist for >60 min, whereas positive selection events may be as short as 15 min.

## Peptide Affinity Has Only a Modest Effect on the Threshold for *Nr4a3* Activation Compared to *Nr4a1*

In order to investigate how expression levels of *Nr4a1* and *Nr4a3* differ in response to different TCR affinities, *Nr4a1*-GFP *Nr4a3*-Tocky mice were crossed with OTI mice. As expected, we observed expression of Nr4a1-GFP, but not Nr4a3-Timer, in TCRβ$^{+}$ DP and CD8SP thymocytes (Figures 5A and 5B). *Nr4a1*-GFP *Nr4a3*-Tocky OTI splenocytes were stimulated with a range of doses of different affinity ova peptides (Figure 5C). Higher affinity peptides N4 (Figure 5D) and Q4 (Figure 5E)

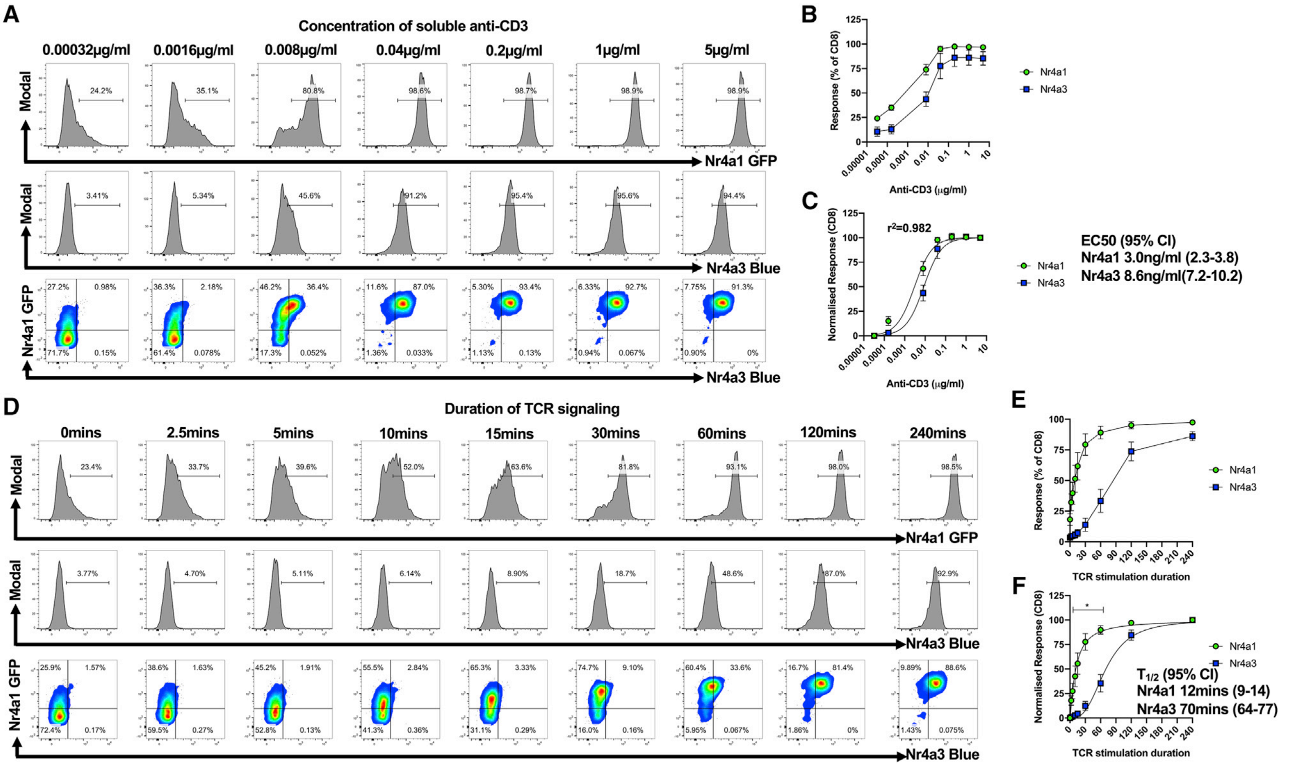


**Figure 4.** ***Nr4a1*** **Is More Sensitive to TCR Signals and Can Be Activated by Shorter TCR Signaling Bursts than** ***Nr4a3***

(A) *Nr4a1*-GFP *Nr4a3*-Tocky splenocytes were cultured with soluble anti-CD3 for 4 h before analysis of live CD8$^+$ T cells for expression of Nr4a1-GFP and Nr4a3-Timer Blue.

(B) Summary showing the raw % of CD8$^+$ T cells in response to anti-CD3; n = 5, bars represent mean ± SEM.

(C) Normalized responses of Nr4a1-GFP and Nr4a3-Timer Blue to anti-CD3; n = 5, bars represent mean ± SD. $EC_{50}$ values and 95% confidence intervals (CIs) are stated.

(D) *Nr4a1*-GFP *Nr4a3*-Tocky splenocytes were stimulated with 5 μg/ml anti-CD3 for 4 h. At the indicated time points, PP2 was added at a final concentration of 10 μM. After 4 h of culture, cells were analyzed for Nr4a1-GFP and Nr4a3-Timer Blue expression in live CD8$^+$ T cells by flow cytometry.

(E) Summary data showing the raw % of CD8$^+$ T cells for Nr4a1-GFP or Nr4a3-Timer Blue in response to the period of TCR signaling; bars represent mean ± SEM, n = 3.

(F) Summary data showing normalized response for Nr4a1-GFP or Nr4a3-Timer Blue in response to the period of TCR signaling; bars represent mean ± SEM, n = 3. *, indicates significant differences ($p < 0.05$) between Nr4a1-GFP and Nr4a3-Timer Blue for 2.5-, 5-, 10-, 15-, 30-, and 60-min time points (analysis by two-way ANOVA with Tukey's multiple comparisons test). Data are pooled from three independent experiments.

triggered expression of both Nr4a1-GFP and Nr4a3-Timer at lower doses. Dose-response curves of Nr4a1-GFP and Nr4a3-Timer expression in response to N4 cognate peptide stimulation demonstrated only very minor differences in $EC_{50}$. In contrast, stimulation with the V4 peptide demonstrated a 3.5-fold difference in $EC_{50}$, and even at saturating doses, <90% of cells were positive for the *Nr4a3* reporter and plateaued before reaching 100% of the normalized response to the N4 peptide (Figure 5F). Taken together, these data demonstrate that *Nr4a1*-GFP and *Nr4a3*-Tocky reporters are regulated by distinct pathways downstream of the TCR and their expression patterns differ due to differential sensitivities to TCR signal strength and duration.

## DISCUSSION

The data have highlighted how the differential sensitivities of *Nr4a1* and *Nr4a3* to distal TCR signaling pathways can be exploited to identify T cells undergoing different types of TCR signaling *in vivo*. *Nr4a1* exhibits a lower threshold for activation in terms of both strength and time required for TCR signaling to elicit its activation. Our data would suggest that Nr4a1-GFP expression can be triggered by short-lived antigen receptor signals, whereas Nr4a3-Timer requires full activation of the NFAT pathway. Nr4a1-GFP expression persists in the periphery, and it is unclear as to what extent this expression is due to residual GFP from selection events in the thymus versus the continued recognition of MHC in the periphery. Given that Tregs continue to express Nr4a3 in the periphery, this would suggest that Tregs continue to undergo signals that cross the threshold for NFAT activation outside the thymus.

Nr4a receptors are emerging as an exciting target for immunomodulation (Flemming, 2019), in particular as a potential strategy to fine-tune CAR T cell attack of solid tumors (Li and Zhang, 2019). Here, we have shown that *Nr4a1*, *Nr4a2*, and *Nr4a3* are distinctly regulated by the CaN/NFAT pathway but

CellPress
OPEN ACCESS

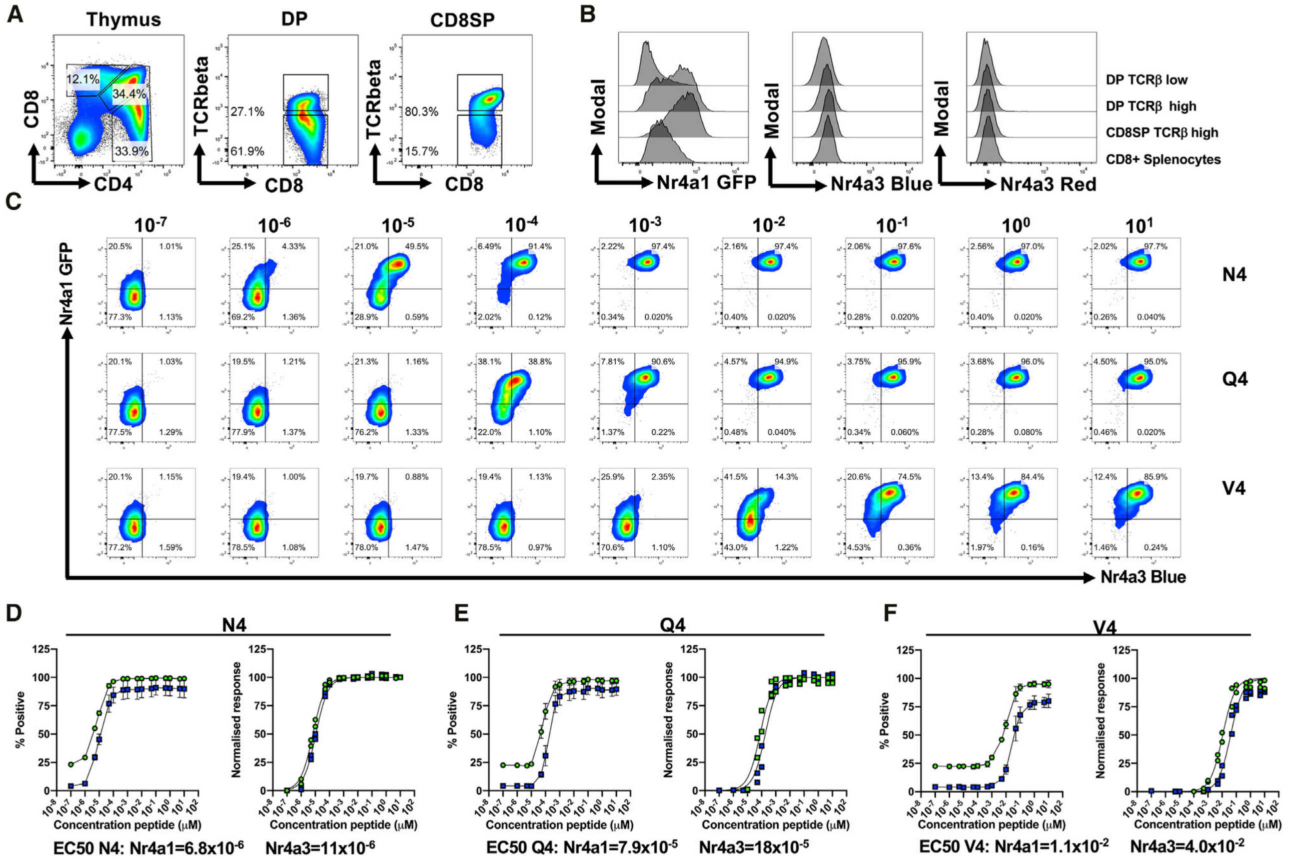

**Figure 5. Peptide Affinity Has Only a Modest Effect on the Threshold for *Nr4a3* Activation Compared to *Nr4a1***

(A) Thymus from OTI *Nr4a1*-GFP *Nr4a3*-Tocky mice displaying CD4 versus CD8 (left), CD8 versus TCR-beta in DP cells (middle), or CD8SP (right) populations.
(B) Histogram overlays of Nr4a1-GFP expression (left), Nr4a3-Timer Blue (middle), or Nr4a3-Timer Red (right) in the thymic or splenic populations indicated.
(C) Splenocytes from OTI *Nr4a1*-GFP *Nr4a3*-Tocky mice were cultured for 5 h in the presence of a dose range of ova peptide variants (concentrations shown are μM scale). N4 (SIINFEKL), Q4 (SIIQFEKL), or V4 (SIIVFEKL). Summary data of N4 (D), Q4 (E), or V4 (F) responses from (C) for Nr4a1-GFP (green) or Nr4a3-Timer Blue (blue) as raw % (bars represent mean ± SEM) of $CD8^+$ T cells (left) or normalized response (right). $EC_{50}$ values are calculated from curve fitting of normalized data. Data are from two independent experiments.

that all three require ERK pathway activation for maximal expression in response to TCR stimulation. Interestingly, an analysis of NR4A1 protein regulation in human T cells has shown that they are also insensitive to CaN inhibitors (Ashouri and Weiss, 2017) but show a partial ERK dependence, suggesting that regulation of *Nr4a1* may be conserved between mice and humans. *Nr4a3* has been shown to be regulated by the MAPK/ERK pathway in response to platelet-derived growth factor in vascular smooth muscle cells (Nomiyama et al., 2006). Similarly, in ovarian cells, calcium-dependent activation of ERK mediates AP-1 induction of *Nr4a1* (Stocco et al., 2002), establishing a common link between ERK signaling and the regulation of *Nr4a* receptor expression across diverse tissue types.

Despite being insensitive to CaN inhibitors, the *Nr4a1* gene region exhibited binding of NFAT1. Surprisingly, both cyclosporin and FK506 had no effect on the transcriptional dynamics of *Nr4a1* expression in response to peptide stimulation of either $CD4^+$ and $CD8^+$ T cells. This finding could represent redundancy in the regulation of *Nr4a1* by NFAT1, given that *Nr4a1* appears more sensitive to a broad range of distal TCR signaling pathways. In particular, it has been previously observed that cyclosporin may interfere with *Nr4a1* biology at the level of its DNA binding activity through its N-terminal protein region (Yazdanbakhsh et al., 1995), without interfering with its transcription. In this way, cyclosporin may abrogate the biological effects of all Nr4a receptors through disparate mechanisms of action. On the other hand, constitutively active NFAT1 did not significantly alter *Nr4a1* expression in $CD4^+$ or $CD8^+$ T cells transduced with the CA-RIT-NFAT1 vector, suggesting that *Nr4a1* cannot be activated by NFAT1 activity alone. However, as the CA-RIT-NFAT1 construct is incapable of binding AP-1 (Martinez et al., 2015), it is still possible that NFAT:AP1 complexes could redundantly activate *Nr4a1* or that NFAT2 and NFAT4 may substitute.

PP2 inhibitor data suggest that *Nr4a* induction is triggered by the activity of lymphocyte-specific protein tyrosine kinase (Lck). Lck associates with the cytoplasmic tails of CD4 and CD8 coreceptors (Veillette et al., 1988). Peptide-MHC engagement of the TCR leads to Lck-mediated phosphorylation of the TCR

and CD3 chains (Straus and Weiss, 1992). Interestingly, past work has suggested that more CD4 co-receptors are loaded than CD8 molecules, which can affect the dwell time of T cells (Stepanek et al., 2014). Our comparison of *Nr4a* transcriptional dynamics suggested that CD8$^+$ T cells receive an initial shorter, sharper, and stronger activation of *Nr4a* transcription, which peaks at 1 h. Interestingly, *Nr4a* receptor transcription in CD4$^+$ T cell peaked around 2 h and appeared to plateau, suggesting potential subtle differences in distal TCR signaling dynamics between CD4$^+$ and CD8$^+$ T cells.

Nr4a1 GFP transgenic reporters (Moran et al., 2011; Zikherman et al., 2012) have been useful tools to study Treg and invariant natural killer T (iNKT) cell development, as well as to address B cell activation to antigen. However, as previously alluded to, temporal analysis of Nr4a1-GFP reporters may be hampered by the persistence of GFP expression following antigen encounter (Au-Yeung et al., 2014). The *Nr4a3*-Tocky system used in this study does not suffer from this same issue because the half-life of the Timer Blue protein is 4 h (Bending et al., 2018b), allowing a sensitive readout over much shorter time frames. Given that NFAT in the absence of AP-1 induces a chronic T cell exhaustion phenotype (Martinez et al., 2015), *Nr4a3*-Tocky will be useful tools for understanding NFAT pathway activity in models of T cell dysfunction and cancer. Furthermore, assessing *Nr4a3* and *Nr4a1* co-regulation will be a potent tool to interrogate alterations in TCR signaling *in vivo*.

The finding that Nr4a1-GFP can be rapidly expressed in response to minutes of TCR signaling suggests that caution should be used when using it as a readout of TCR signal strength; moreover, an accumulation of GFP appears to be an event that is time dependent, and therefore, different levels of GFP may reflect different durations of TCR signals. Indeed, an analysis of mice using the markers CD5, Ly6C, and Nr4a1-GFP have shown that a broad range exists *in vivo* for naive T cells even within the same TCR niche (Zinzow-Kramer et al., 2019). These findings suggest that conventional T cells likely continue to receive sporadic short-lived signals in the periphery but that Tregs undergo full and frequent NFAT pathway activation.

A potential limitation of the study is that we cannot exclude the possibility that differential sensitivity of flow cytometers to detect GFP versus Timer protein could contribute to the failure to detect T cells expressing low levels of *Nr4a3*. It is possible that the variation in BAC copy number between the systems could also influence the sensitivity of the reporters. In order to test this, *Nr4a1* and *Nr4a3* BAC GFP reporters with known copy numbers would need to be compared head to head. However, in terms of quantum yield, calculations for Timer Blue are in the range 0.30–0.41 (Subach et al., 2009), which is comparable to EGFP (0.60; Ilagan et al., 2010). Additionally, in our comparison of signal duration, for which we pharmacologically inhibited TCR signaling (Figure 4D), we left the cells in culture for 4 h to allow for differences in folding and fluorescence of the proteins. This means that we maximized our ability to detect the presence of any fluorescent proteins that would have accumulated in response to the initial signal.

In summary, our findings highlight the distinct sensitivity of *Nr4a* family members to the NFAT pathway. Shorter and weaker TCR signals elicit Nr4a1-GFP expression in response to TCR signaling during lymphocyte development. Sustained TCR signaling is required for Nr4a3-Timer expression, highlighting that signal duration should also be considered in relation to signal strength when interpreting TCR signaling using *Nr4a* receptor reporter mice.

## STAR★METHODS

Detailed methods are provided in the online version of this paper and include the following:


- KEY RESOURCES TABLE
- RESOURCE AVAILABILITY
  - Lead Contact
  - Materials Availability
  - Data and Code Availability
- EXPERIMENTAL MODEL AND SUBJECT DETAILS
  - *Nr4a3*-Tocky Mice
  - Tg4 TCR Transgenic Mice
  - IL-10-GFP Tiger Mice
  - Great-Smart17A Mice
  - Nr4a1/Nur77-GFP Mice
  - OTI Mice
  - Breeding
- METHOD DETAILS
  - *In Vitro* Cultures
  - Flow cytometric analysis
  - *In Vivo* Immunisation
  - qPCR analysis
  - *In silico* ChIP-Seq
  - RNaseq analysis
  - CD4 and CD8 purification
- QUANTIFICATION AND STATISTICAL ANALYSIS


## SUPPLEMENTAL INFORMATION

Supplemental Information can be found online at https://doi.org/10.1016/j.celrep.2020.108328.

## ACKNOWLEDGMENTS

This work was funded by the Wellcome Trust Seed award (D.B., E.J., and T.A.E.E.; 214018/Z/18/Z). S.K. was supported by an Arthur Thomson summer studentship. M.O. was supported by a BBSRC David Phillips Fellowship (BB/J013951/1). D.C.W. is supported by the University of Birmingham. D.B. is supported by a University of Birmingham Fellowship. K.-M.T. and J.C.Y.-P. were supported by the BBSRC (BB/S003800/1). G.A. is supported by an MRC Programme Grant (MR/N000919/1). The graphical abstract was created with BioRender.com.

## AUTHOR CONTRIBUTIONS

D.B. designed and conceptualized the experiments, performed *in silico* analyses, and together with T.A.E.E. wrote the paper. E.J., T.A.E.E., N.T., and S.K. conducted experiments. D.C.W. provided the Tg4 Tiger mouse line, and M.O. provided the *Nr4a3*-Tocky strain. G.A. provided the *Nr4a1*/Nur77-GFP mouse line and expert advice on thymic T cell development. J.C.Y.-P. and K.-M.T. provided reagents and expert advice on flow cytometric staining for B cell development.

## DECLARATION OF INTERESTS

The authors declare no competing interests.

CellPress
OPEN ACCESS

Received: October 7, 2019
Revised: August 7, 2020
Accepted: October 8, 2020
Published: November 3, 2020

## REFERENCES

Ashouri, J.F., and Weiss, A. (2017). Endogenous Nur77 Is a Specific Indicator of Antigen Receptor Signaling in Human T and B Cells. J. Immunol. *198*, 657–668.

Au-Yeung, B.B., Zikherman, J., Mueller, J.L., Ashouri, J.F., Matloubian, M., Cheng, D.A., Chen, Y., Shokat, K.M., and Weiss, A. (2014). A sharp T-cell antigen receptor signaling threshold for T-cell proliferation. Proc. Natl. Acad. Sci. USA *111*, E3679–E3688.

Barrett, S.D., Bridges, A.J., Dudley, D.T., Saltiel, A.R., Fergus, J.H., Flamme, C.M., Delaney, A.M., Kaufman, M., LePage, S., Leopold, W.R., et al. (2008). The discovery of the benzhydroxamate MEK inhibitors CI-1040 and PD 0325901. Bioorg. Med. Chem. Lett. *18*, 6501–6504.

Bending, D., Paduraru, A., Ducker, C.B., Prieto Martín, P., Crompton, T., and Ono, M. (2018a). A temporally dynamic *Foxp3* autoregulatory transcriptional circuit controls the effector Treg programme. EMBO J. *37*, e99013.

Bending, D., Prieto Martín, P., Paduraru, A., Ducker, C., Marzaganov, E., Laviron, M., Kitano, S., Miyachi, H., Crompton, T., and Ono, M. (2018b). A timer for analyzing temporally dynamic changes in transcription during differentiation in vivo. J. Cell Biol. *217*, 2931–2950.

Brownlie, R.J., and Zamoyska, R. (2013). T cell receptor signalling networks: branched, diversified and bounded. Nat. Rev. Immunol. *13*, 257–269.

Burton, B.R., Britton, G.J., Fang, H., Verhagen, J., Smithers, B., Sabatos-Peyton, C.A., Carney, L.J., Gough, J., Strobel, S., and Wraith, D.C. (2014). Sequential transcriptional changes dictate safe and effective antigen-specific immunotherapy. Nat. Commun. *5*, 4741.

Chen, J., López-Moyado, I.F., Seo, H., Lio, C.J., Hempleman, L.J., Sekiya, T., Yoshimura, A., Scott-Browne, J.P., and Rao, A. (2019). NR4A transcription factors limit CAR T cell function in solid tumours. Nature *567*, 530–534.

Cheng, L.E., Chan, F.K., Cado, D., and Winoto, A. (1997). Functional redundancy of the Nur77 and Nor-1 orphan steroid receptors in T-cell apoptosis. EMBO J. *16*, 1865–1875.

Fassett, M.S., Jiang, W., D'Alise, A.M., Mathis, D., and Benoist, C. (2012). Nuclear receptor Nr4a1 modulates both regulatory T-cell (Treg) differentiation and clonal deletion. Proc. Natl. Acad. Sci. USA *109*, 3891–3896.

Flemming, A. (2019). What's driving T cell dysfunction? Nat. Rev. Immunol. *19*, 199.

Hanke, J.H., Gardner, J.P., Dow, R.L., Changelian, P.S., Brissette, W.H., Weringer, E.J., Pollok, B.A., and Connelly, P.A. (1996). Discovery of a novel, potent, and Src family-selective tyrosine kinase inhibitor. Study of Lck- and FynT-dependent T cell activation. J. Biol. Chem. *271*, 695–701.

Hibino, S., Chikuma, S., Kondo, T., Ito, M., Nakatsukasa, H., Omata-Mise, S., and Yoshimura, A. (2018). Inhibition of Nr4a Receptors Enhances Antitumor Immunity by Breaking Treg-Mediated Immune Tolerance. Cancer Res. *78*, 3027–3040.

Hogan, P.G., Chen, L., Nardone, J., and Rao, A. (2003). Transcriptional regulation by calcium, calcineurin, and NFAT. Genes Dev. *17*, 2205–2232.

Hogquist, K.A., Jameson, S.C., Heath, W.R., Howard, J.L., Bevan, M.J., and Carbone, F.R. (1994). T cell receptor antagonist peptides induce positive selection. Cell *76*, 17–27.

Ilagan, R.P., Rhoades, E., Gruber, D.F., Kao, H.T., Pieribone, V.A., and Regan, L. (2010). A new bright green-emitting fluorescent protein—engineered monomeric and dimeric forms. FEBS J. *277*, 1967–1978.

Jain, J., Miner, Z., and Rao, A. (1993). Analysis of the preexisting and nuclear forms of nuclear factor of activated T cells. J. Immunol. *151*, 837–848.

Kamanaka, M., Kim, S.T., Wan, Y.Y., Sutterwala, F.S., Lara-Tejero, M., Galán, J.E., Harhaj, E., and Flavell, R.A. (2006). Expression of interleukin-10 in intestinal lymphocytes detected by an interleukin-10 reporter knockin tiger mouse. Immunity *25*, 941–952.

Karin, M. (1995). The regulation of AP-1 activity by mitogen-activated protein kinases. J. Biol. Chem. *270*, 16483–16486.

Li, F., and Zhang, Y. (2019). Targeting NR4As, a new strategy to fine-tune CAR-T cells against solid tumors. Signal Transduct. Target. Ther. *4*, 7.

Liebmann, M., Hucke, S., Koch, K., Eschborn, M., Ghelman, J., Chasan, A.I., Glander, S., Schädlich, M., Kuhlencord, M., Daber, N.M., et al. (2018). Nur77 serves as a molecular brake of the metabolic switch during T cell activation to restrict autoimmunity. Proc. Natl. Acad. Sci. USA *115*, E8017–E8026.

Liu, J., Farmer, J.D., Jr., Lane, W.S., Friedman, J., Weissman, I., and Schreiber, S.L. (1991). Calcineurin is a common target of cyclophilin-cyclosporin A and FKBP-FK506 complexes. Cell *66*, 807–815.

Liu, Z.G., Smith, S.W., McLaughlin, K.A., Schwartz, L.M., and Osborne, B.A. (1994). Apoptotic signals delivered through the T-cell receptor of a T-cell hybrid require the immediate-early gene nur77. Nature *367*, 281–284.

Liu, G.Y., Fairchild, P.J., Smith, R.M., Prowle, J.R., Kioussis, D., and Wraith, D.C. (1995). Low avidity recognition of self-antigen by T cells permits escape from central tolerance. Immunity *3*, 407–415.

Liu, X., Wang, Y., Lu, H., Li, J., Yan, X., Xiao, M., Hao, J., Alekseev, A., Khong, H., Chen, T., et al. (2019). Genome-wide analysis identifies NR4A1 as a key mediator of T cell dysfunction. Nature *567*, 525–529.

Macian, F. (2005). NFAT proteins: key regulators of T-cell development and function. Nat. Rev. Immunol. *5*, 472–484.

Martinez, G.J., Pereira, R.M., Äijö, T., Kim, E.Y., Marangoni, F., Pipkin, M.E., Togher, S., Heissmeyer, V., Zhang, Y.C., Crotty, S., et al. (2015). The transcription factor NFAT promotes exhaustion of activated $CD8^+$ T cells. Immunity *42*, 265–278.

Matsuda, S., and Koyasu, S. (2000). Mechanisms of action of cyclosporine. Immunopharmacology *47*, 119–125.

Mognol, G.P., Spreafico, R., Wong, V., Scott-Browne, J.P., Togher, S., Hoffmann, A., Hogan, P.G., Rao, A., and Trifari, S. (2017). Exhaustion-associated regulatory regions in $CD8^+$ tumor-infiltrating T cells. Proc. Natl. Acad. Sci. USA *114*, E2776–E2785.

Moran, A.E., Holzapfel, K.L., Xing, Y., Cunningham, N.R., Maltzman, J.S., Punt, J., and Hogquist, K.A. (2011). T cell receptor signal strength in Treg and iNKT cell development demonstrated by a novel fluorescent reporter mouse. J. Exp. Med. *208*, 1279–1289.

Nomiyama, T., Nakamachi, T., Gizard, F., Heywood, E.B., Jones, K.L., Ohkura, N., Kawamori, R., Conneely, O.M., and Bruemmer, D. (2006). The NR4A orphan nuclear receptor NOR1 is induced by platelet-derived growth factor and mediates vascular smooth muscle cell proliferation. J. Biol. Chem. *281*, 33467–33476.

Peterson, B.R., Sun, L.J., and Verdine, G.L. (1996). A critical arginine residue mediates cooperativity in the contact interface between transcription factors NFAT and AP-1. Proc. Natl. Acad. Sci. USA *93*, 13671–13676.

Price, A.E., Reinhardt, R.L., Liang, H.E., and Locksley, R.M. (2012). Marking and quantifying IL-17A-producing cells in vivo. PLoS One *7*, e39750.

Scott-Browne, J.P., López-Moyado, I.F., Trifari, S., Wong, V., Chavez, L., Rao, A., and Pereira, R.M. (2016). Dynamic Changes in Chromatin Accessibility Occur in $CD8^+$ T Cells Responding to Viral Infection. Immunity *45*, 1327–1340.

Sekiya, T., Kashiwagi, I., Inoue, N., Morita, R., Hori, S., Waldmann, H., Rudensky, A.Y., Ichinose, H., Metzger, D., Chambon, P., and Yoshimura, A. (2011). The nuclear orphan receptor Nr4a2 induces Foxp3 and regulates differentiation of CD4+ T cells. Nat. Commun. *2*, 269.

Sekiya, T., Kashiwagi, I., Yoshida, R., Fukaya, T., Morita, R., Kimura, A., Ichinose, H., Metzger, D., Chambon, P., and Yoshimura, A. (2013). Nr4a receptors are essential for thymic regulatory T cell development and immune homeostasis. Nat. Immunol. *14*, 230–237.

Seo, H., Chen, J., González-Avalos, E., Samaniego-Castruita, D., Das, A., Wang, Y.H., López-Moyado, I.F., Georges, R.O., Zhang, W., Onodera, A., et al. (2019). TOX and TOX2 transcription factors cooperate with NR4A

transcription factors to impose CD8$^+$ T cell exhaustion. Proc. Natl. Acad. Sci. USA *116*, 12410–12415.

Shimizu, K., Sugiura, D., Okazaki, I.M., Maruhashi, T., Takegami, Y., Cheng, C., Ozaki, S., and Okazaki, T. (2020). PD-1 Imposes Qualitative Control of Cellular Transcriptomes in Response to T Cell Activation. Mol. Cell *77*, 937–950.e936.

Stepanek, O., Prabhakar, A.S., Osswald, C., King, C.G., Bulek, A., Naeher, D., Beaufils-Hugot, M., Abanto, M.L., Galati, V., Hausmann, B., et al. (2014). Coreceptor scanning by the T cell receptor provides a mechanism for T cell tolerance. Cell *159*, 333–345.

Stocco, C.O., Lau, L.F., and Gibori, G. (2002). A calcium/calmodulin-dependent activation of ERK1/2 mediates JunD phosphorylation and induction of nur77 and 20alpha-hsd genes by prostaglandin F2alpha in ovarian cells. J. Biol. Chem. *277*, 3293–3302.

Straus, D.B., and Weiss, A. (1992). Genetic evidence for the involvement of the lck tyrosine kinase in signal transduction through the T cell antigen receptor. Cell *70*, 585–593.

Subach, F.V., Subach, O.M., Gundorov, I.S., Morozova, K.S., Piatkevich, K.D., Cuervo, A.M., and Verkhusha, V.V. (2009). Monomeric fluorescent timers that change color from blue to red report on cellular trafficking. Nat. Chem. Biol. *5*, 118–126.

Thompson, J., and Winoto, A. (2008). During negative selection, Nur77 family proteins translocate to mitochondria where they associate with Bcl-2 and expose its proapoptotic BH3 domain. J. Exp. Med. *205*, 1029–1036.

Thomson, A.W., Bonham, C.A., and Zeevi, A. (1995). Mode of action of tacrolimus (FK506): molecular and cellular mechanisms. Ther. Drug Monit. *17*, 584–591.

Veillette, A., Bookman, M.A., Horak, E.M., and Bolen, J.B. (1988). The CD4 and CD8 T cell surface antigens are associated with the internal membrane tyrosine-protein kinase p56lck. Cell *55*, 301–308.

Wang, Z., Benoit, G., Liu, J., Prasad, S., Aarnisalo, P., Liu, X., Xu, H., Walker, N.P., and Perlmann, T. (2003). Structure and function of Nurr1 identifies a class of ligand-independent nuclear receptors. Nature *423*, 555–560.

Yazdanbakhsh, K., Choi, J.W., Li, Y., Lau, L.F., and Choi, Y. (1995). Cyclosporin A blocks apoptosis by inhibiting the DNA binding activity of the transcription factor Nur77. Proc. Natl. Acad. Sci. USA *92*, 437–441.

Zikherman, J., Parameswaran, R., and Weiss, A. (2012). Endogenous antigen tunes the responsiveness of naive B cells but not T cells. Nature *489*, 160–164.

Zinzow-Kramer, W.M., Weiss, A., and Au-Yeung, B.B. (2019). Adaptation by naïve CD4$^+$ T cells to self-antigen-dependent TCR signaling induces functional heterogeneity and tolerance. Proc. Natl. Acad. Sci. USA *116*, 15160–15169.

## STAR★METHODS

### KEY RESOURCES TABLE

| REAGENT or RESOURCE | SOURCE | IDENTIFIER |
|---|---|---|
| Antibodies | | |
| Anti CD4 AF700 Rat monoclonal RM4-4 | BioLegend | Cat# 116022; RRID: AB_2715957 |
| Anti CD4 BUV737 Rat monoclonal GK1.5 | BDBiosciences | Cat# 564298; RRID: AB_2738734 |
| Anti TCRVβ 8.1/8.2 PerCP-eFluor710 Rat monoclonal KJ16-133 | ThermoFisher | Cat# 46-5813-82; RRID: AB_10549113 |
| Anti TCRVβ 8.1/8.2 BUV395 Rat monoclonal MR5-2 | BDBiosciences | Cat# 744335; RRID: AB_2742163 |
| Anti TCRβ AF700 Armenian Hamster monoclonal H57-597 | BioLegend | Cat# 109223; RRID: AB_109223 |
| Anti CD69 APC Armenian Hamster monoclonal H1.2F3 | BioLegend | Cat# 104513; RRID: AB_492844 |
| Anti CD8 AF700 Rat monoclonal 53-6.7 | BioLegend | Cat# 100729; RRID: AB_493702 |
| Anti CD8 BUV395 Rat monoclonal 53-6.7 | BDBiosciences | Cat# 563786; RRID: AB_2732919 |
| Anti TCR Vα2 PerCP/Cyanine5.5 Rat monoclonal B20.1 | BioLegend | Cat# 127813; RRID: AB_1186118 |
| Anti TCR Vβ5.1/5.2 APC Mouse monoclonal MR9-4 | BioLegend | Cat# 139505; RRID: AB_10897800 |
| Anti B220 PerCP-Cy5.5 Rat monoclonal RA3-6B2 | BDBiosciences | Cat# 561101; RRID: AB_10565970 |
| Anti CD43 Biotin Rat monoclonal S7 | BDBiosciences | Cat# 553269; RRID: AB_2255226 |
| Anti IgM PE-Cy7 Rat monoclonal RMM-1 | BioLegend | Cat# 406513; RRID AB_10640069 |
| Anti IgD APC Rat monoclonal 11-26C | Invitrogen | Cat# 17-5993-82; RRID: AB_10598660 |
| Anti CD21 APC Rat monoclonal 7G6 | BDBiosciences | Cat# 561770; RRID: AB_10892818 |
| Anti CD23 PE-Cy7 Rat monoclonal B3B4 | ThermoFisher | Cat# 25-0232-82; RRID: AB_469604 |
| Anti CD25 PerCP/Cy5.5 Rat monoclonal PC61 | BioLegend | Cat# 102029; RRID: AB_893291 |
| Anti CD19 BUV737 Rat monoclonal 1D3 | BDBiosciences | Cat# 564296; RRID: AB_2716855 |
| Anti CD3ε Armenian Hamster monoclonal 145-2C11 | BioLegend | Cat# 100301; RRID: AB_312666 |
| Chemicals, Peptides, and Recombinant Proteins | | |
| Streptavidin BV711 | BioLegend | Cat# 405241 |
| MBP Ac1-9[4Y] peptide AcASQYRPSQR | GL Biochem Shanghai | Custom product |
| N4 OVA 257-264 SIINFEKL peptide | GL Biochem Shanghai | Cat# 181660 |
| Q4 OVA 257-264 SIIQFEKL peptide | GL Biochem Shanghai | Cat# 151560 |
| V4 OVA 257-264 SIIVFEKL peptide | GL Biochem Shanghai | Cat# 151561 |
| Cyclosporin A | Cell Guidance Systems | Cat# SM43; CAS: 59865-13-3 |
| FK506 (Tacrolimus) | Cayman Chemical | Cat# 10007965; CAS: 59865-13-3 |
| PD 0325901 | Cayman Chemical | Cat# 13034; CAS: 391210-10-9 |
| DMSO | Sigma Aldrich | Cat# D2650; CAS: 67-68-5 |
| PP2 | Sigma Aldrich | Cat# P0042; CAS: 172889-27-9 |
| Critical Commercial Assays | | |
| RNeasy mini kit | QIAGEN | Cat# 74104 |
| Invitrogen Superscript IV Reverse Transcriptase | ThermoFisher | Cat# 18090050 |
| Invitrogen Random hexamers | ThermoFisher | Cat# N8080127 |
| Applied Biosystems SYBR green power up master mix | ThermoFisher | Cat# A25752 |
| eFluor-780 fixable viability dye | eBioscience | Cat# 65-0865-14 |
| MoJo Sort nanobeads: CD8 T Cell Isolation Kit | BioLegend | Cat# 480035 |
| MoJo Sort nanobeads: naive CD4 T Cell Isolation Kit | BioLegend | Cat# 480039 |

(*Continued on next page*)

*Continued*

| REAGENT or RESOURCE | SOURCE | IDENTIFIER |
|---|---|---|
| Deposited Data | | |
| RNA-seq of CA-RIT-NFAT1 CD8 T cells, NFAT1 ChIP-seq of WT and NFAT−/− CD8 T cells | Martinez et al., 2015 | GEO: GSE64409 |
| Experimental Models: Organisms/Strains | | |
| Mouse: *Nr4a3*-Tocky founder line 323 | Bending et al., 2018b; Obtained from Dr. Masahiro Ono from Imperial College London under MTA | PMID: 29941474 |
| Mouse:Tg4-H2$^{U}$ | Liu et al., 1995; Provided by Prof. David Wraith University of Birmingham | PMID: 7584132 |
| Mouse: IL-10-GFP Tiger | Kamanaka et al., 2006; Provided by Prof. David Wraith University of Birmingham | PMID: 17137799 |
| Mouse: Great-Smart17A | Price et al., 2012; Obtained from Prof. Richard Locksley from University of California, San Francisco under MTA | PMID: 22768117 |
| Mouse: Nr4a1/Nur77-GFP | Moran et al., 2011; Provided by Prof. Graham Anderson University of Birmingham | PMID: 21606508 |
| Mouse: OTI | Charles River Laboratories | Strain Code: 642 |
| Oligonucleotides | | |
| *Hprt* for: AGCCTAAGATGAGCGCAAGT rev: TTACTAGGCAGATGGCCACA | Bending et al., 2018a | PMID: 29991564 |
| *Nr4a1* for: TGTGAGGGCTGCAAGGGCTTC rev: AAGCGGCAGAACTGGCAGCGG | Sekiya et al., 2013 | PMID: 23334790 |
| *Nr4a2* for: CTGTGCGCTGTTTGCGGTGAC rev: CGGCGCTTGTCCACTGGGCAG | Sekiya et al., 2013 | PMID: 23334790 |
| *Nr4a3* for: AGGGCTTCTTCAAGAGAACGG rev: CCATCCCGACACTGAGACAC | This paper, Designed using NCBI Primer Blast | N/A |
| Software and Algorithms | | |
| GraphPad Prism 7 and 8 | | https://www.graphpad.com/scientific-software/prism/ |
| FlowJo v10.5.3 | | https://www.flowjo.com/solutions/flowjo |
| CyVerse Discovery Environment | | https://cyverse.org/discovery-environment |
| UCSC genome browser | | https://genome.ucsc.edu |

## RESOURCE AVAILABILITY

### Lead Contact

Further information and requests for resource and reagents should be directed to and will be fulfilled by the Lead Contact Dr. David Bending (d.a.bending@bham.ac.uk).

### Materials Availability

This study did not generate new unique reagents. *Nr4a3*-Tocky mice are available from Dr. M. Ono, Imperial College London, under material transfer agreement (MTA). Great Smart-17A mice are available from Prof. R. Locksley, UCSF under MTA.

### Data and Code Availability

This article includes analysis of previously published datasets: RNA-seq of CA-RIT-NFAT1 CD8 T cells, NFAT1 ChIP-seq of WT and NFAT1−/− CD8 T cells – GEO: GSE64409, (Martinez et al., 2015).

## EXPERIMENTAL MODEL AND SUBJECT DETAILS

All animal experiments were performed in accordance with local Animal Welfare and Ethical Review Body at the University of Birmingham and under the authority of a Home Office project license, P18A892E0A held by D.B. Animals were housed in specific pathogen-free conditions with appropriate housing conditions and husbandry as specified by NC3Rs. Genotypes of transgenic mice were confirmed by end point PCR of ear skin samples and agarose gel electrophoresis. Male and female mice were used, littermates of the same sex were randomly assigned to experimental groups.

### *Nr4a3*-Tocky Mice

*Nr4a3*-Tocky mice expressing a BAC containing FT Fast mCherry mutant (Subach et al., 2009) under the influence of *Nr4a3* regulatory regions on the C57BL/6J background as previously described (Bending et al., 2018b), were obtained from Dr. Masahiro Ono, Imperial College London, under MTA.

### Tg4 TCR Transgenic Mice

Tg4 mice expressing the αβ TCR (Vα4, Vβ8.2) of the MBP Ac1-9-specific hybridoma 1934.4 (Liu et al., 1995).

### IL-10-GFP Tiger Mice

IL-10-GFP Tiger mice expressing an IRES *GFP* transgene inserted into the *Il10* locus on the C57BL/6 background as previously described (Kamanaka et al., 2006).

### Great-Smart17A Mice

Great-Smart17A expressing an IRES *YFP* transgene inserted into the *Ifng* locus and an IRES *hNGFR* transgene inserted into the *Il17A* locus as previously described (Price et al., 2012). Great-Smart-17A mice were obtained from Prof. Richard Locksley, UCSF, under MTA.

### Nr4a1/Nur77-GFP Mice

Nr4a1/Nur77-GFP mice expressing a BAC containing *GFP* transgene under the influence of *Nr4a1* regulatory regions on C57BL/6J background as previously described (Moran et al., 2011). Nur77-GFP mice were provided by Prof. Graham Anderson, University of Birmingham.

### OTI Mice

OTI mice expressing the αβ TCR (Vα2, Vβ5) of the $OVA_{257\text{-}264}$-specific CTL clone 149.42 as previously described (Hogquist et al., 1994). OTI mice were purchased from Charles River Laboratories.

### Breeding

*Nr4a3*-Tocky Tg4 Tiger mice were used as the F1 generation by crossing *Nr4a3*-Tocky mice with Tg4 IL-10-GFP Tiger (Burton et al., 2014), which were provided by Prof. David Wraith, University of Birmingham, UK. *Nr4a3*-Tocky Great-Smart17A were initially bred to homozygous OT1 mice to generate F1 OT1 *Nr4a3*-Tocky Great Smart-17A mice. Nr4a1-GFP mice were used alone or bred to Nr4a3-Tocky mice to generate *Nr4a1*-GFP *Nr4a3*-Tocky. *Nr4a3*-Tocky zygosity was determined based on mendelian inheritance or by phenotype of Nr4a3-Timer expression by flow cytometry.

## METHOD DETAILS

### *In Vitro* Cultures

Single cell suspensions of splenocytes from *Nr4a3*-Tocky Tg4 Tiger and OTI *Nr4a3*-Tocky mice were generated by forcing organs through 70-μm cell strainers (Corning). For spleens, a red blood cell (RBC) lysis stage was performed (Invitrogen) according to manufacturer's instructions. Cells were washed once and cultured at $1 \times 10^6$ cells per well on 96-well U-bottom plates (Corning) with or without the presence of peptides or anti-CD3 (145-2C11, BioLegend) in a final volume of 200 μl RPMI1640 + L-glutamine (GIBCO) containing 10% FCS and 1% penicillin/streptomycin (Life Technologies). Inhibitors were dissolved in DMSO. The following inhibitors were dissolved in DMSO (Sigma) and used: Cyclosporin A (Cambridge Bioscience, 1 μM or as stated), FK506 (Cayman Chemical Company, 1 μM), PD0325901 (Cambridge Bioscience, 5 μM), PP2 (Sigma, 10 μM) and DMSO (Sigma, 0.1%). In experiments were inhibitors were used, cells were pre-incubated with inhibitors at indicated doses for 30 minutes unless otherwise specified. For Tg4 stimulation 10 μM of MBP Ac1-9[4Y] peptide was used, for OTI stimulation 1 μM peptide $OVA_{257\text{-}264}$ (N4 = SIINFELK, Q4 = SIIQFEKL4, V4 = SIIVFEKL variants as indicated) was used, unless stated. For soluble anti-CD3 mediated stimulation, 5 μg/ml soluble anti-CD3 was used. Cells were incubated at 37°C and 5% CO2 and analyzed at the indicated time points for RNA expression or flow cytometric analysis. For termination of TCR signal studies in Figures 5D–5F, splenocytes from *Nr4a1*-GFP *Nr4a3*-Tocky mice were cultured for 4 hrs with 5 μg/ml anti-CD3. At 2.5-, 5-, 10-, 15-, 30-, 60-, 120-minutes PP2 (Sigma) was added at a final concentration of 10 μM. Cells remained in the incubator until 240 mins from addition of anti-CD3 before harvesting for analysis of Nr4a1-GFP and Nr4a3-Blue expression.

### Flow cytometric analysis

For analysis of thymus and splenic lymphocytes single cell suspensions were prepared as described above. For analysis of bone marrow B cells, femurs were flushed with media and then RBC lysis performed before staining for flow cytometric analysis. Cells were washed once and stained in 96-well U-bottom plates (Corning). Analysis was performed on a BD LSR Fortessa X-20 instrument. The blue form of the Timer protein was detected in the blue (450/40 nm) channel excited off the 405 nm laser. The red form of the Timer protein was detected in the mCherry (610/20) channel excited off the 561 nm laser. A fixable eFluor 780-flurescent viability dye (eBioscience) was used for all experiments. The following antibodies were used: anti-CD4 AF700, anti-TCRβ AF700, anti-CD69 APC, anti-CD8 AF700, anti TCR Vα2 PerCP/Cyanine5.5, anti-TCR Vβ5.1/5.2 APC, Streptavidin BV711, anti-IgM PE-Cy7, and anti-CD25 PerCP/Cy5.5 (all from BioLegend). Anti-IgD APC was from Invitrogen. Anti-CD23 PE-Cy7 and anti-TCRvβ8.1/8.2 PerCP-eFluor710 were from ThermoFisher. Anti-CD19 BUV737, anti-CD4 BUV737, anti-B220 PerCP-Cy5.5, anti-CD21 APC, anti-CD43 Biotin, anti-TCRvβ8.1/8.2 BUV395 and anti-CD8 BUV395 were all from BD Biosciences.

### *In Vivo* Immunisation

Tg4 *Nr4a3*-Tocky mice were immunized with 80 μg of MBP Ac1-9[4Y], AcASQYRPSQR, peptide or vehicle control (PBS) via subcutaneous injection. 4 hours later, mice were culled and the thymus was removed for flow cytometric analysis.

### qPCR analysis

Following *in vitro* cultures, RNA was extracted using RNeasy mini kit (QIAGEN) according to manufacturer's instructions. cDNA was generated using random hexamers (ThermoFisher) and Superscript IV reverse transcriptase (ThermoFisher) according to manufacturer's instructions. mRNA expression was quantified using PowerUp SYBR green (ThermoFisher) and normalized to housekeeping gene *Hprt* using 7900HT sequence detection system or Applied Biosystems AB7500 system. Fold change in expression was calculated using the delta-deltaCt method. Primer sequences: *Hprt* for: AGCCTAAGATGAGCGCAAGT rev: TTACTAGGCAGATGGCCACA; *Nr4a1* for: TGTGAGGGCTGCAAGGGCTTC rev: AAGCGGCAGAACTGGCAGCGG; *Nr4a2* for: CTGTGCGCTGTTTGCGGTGAC rev: CGGCG CTTGTCCACTGGGCAG, *Nr4a3* for: AGGGCTTCTTCAAGAGAACGG rev: CCATCCCGACACTGAGACAC.

### *In silico* ChIP-Seq

Processed bigwig data files deposited in GEO: GSE64409 (Martinez et al., 2015) were downloaded and hosted in CyVerse Discovery Environment (https://de.cyverse.org/de) and then mapped against the mm9 genome using the UCSC genome browser. These files contain analysis of NFAT1 ChIP-Seq in CD8 T cells from WT and NFAT1KO mice either unstimulated or stimulated with PMA and Ionomycin as described in Martinez et al. (2015).

### RNaseq analysis

Log2 fold change estimates of *Nr4a1*, *Nr4a2*, *Nr4a3* and Cd69 expression was extracted from DESeq data deposited in GEO: GSE64409 (Martinez et al., 2015) for CD4+ or CD8+ T cells either transfected with mock vector or CA-RIT-NFAT1.

### CD4 and CD8 purification

Naive CD4 or bulk CD8 T cell populations were negatively selected using MoJo naive murine CD4 T cell isolation kit (BioLegend) or murine CD8 T cell isolation kit (BioLegend) by immunomagnetic selection according to the manufacturer's instructions.

## QUANTIFICATION AND STATISTICAL ANALYSIS

Statistical analysis was performed on Prism 7 or 8 software (GraphPad). For comparison of more than two means a one-way ANOVA with Turkey's multiple comparisons test was used. For comparison of more than two means over time, a two-way ANOVA with Tukey's multiple comparison's test was used. Curve fit analysis using non-linear fitting using the normalized response and slope function on prism 8 software. Variance is reported as mean ± SEM unless otherwise stated. P values that are indicated graphically represent: *p = < 0.05, **p < 0.01, ***p < 0.001, ****p = < 0.0001. N values can be found within the figure legends and indicate number of animals. Adjusted p values for analysis of RNA-seq data deposited by Martinez et al. (2015) were extracted from the DESeq analysis provided by the reference GEO: GSE64409.

**Cell Reports, Volume *33***

## Supplemental Information

# Nr4a1 and Nr4a3 Reporter Mice Are Differentially Sensitive to T Cell Receptor Signal Strength and Duration

**Emma Jennings, Thomas A.E. Elliot, Natasha Thawait, Shivani Kanabar, Juan Carlos Yam-Puc, Masahiro Ono, Kai-Michael Toellner, David C. Wraith, Graham Anderson, and David Bending**

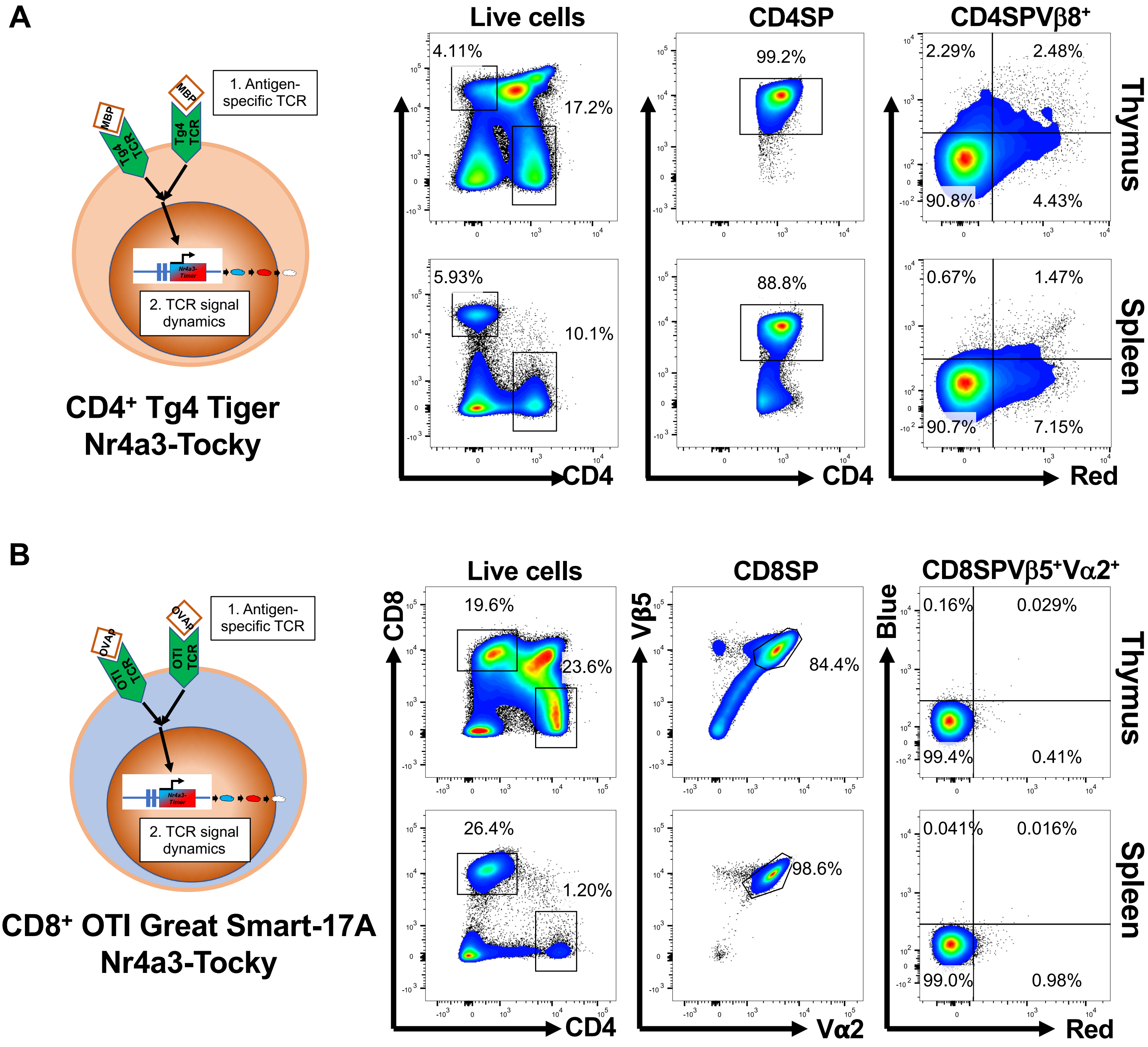


**Supplementary Figure 1: Analysis of Tg4 Nr4a3-Tocky and OTI Nr4a3-Tocky mice (related to Figure 1)**

**(A)** Nr4a3-Tocky mice were crossed with Tg4 Tiger mice to generate Tg4 Tiger Nr4a3-Tocky mice, which are specific for myelin basic protein-derived peptides. Thymus (top) and spleen (bottom) were analysed for CD4 vs. CD8 expression within live lymphocytes (left), CD4 vs. TCRVβ8.1/8.2 (middle) gated on CD4SP cells, and Nr4a3-Timer Blue vs Nr4a3-Timer Red in CD4SPVβ8.1/8.2$^{+}$ T cells. **(B)** Nr4a3-Tocky Great Smart-17A mice were bred with OTI TCR transgenic mice, which are specific for ova peptide. Thymus (top) and spleen (bottom) were analysed for CD4 vs CD8 expression within live lymphocytes (left), TCRVβ5.1/5.2 vs TCRVα2 (middle) gated on CD8SP cells, and Nr4a3-Timer Blue vs Nr4a3-Timer Red in CD8SPVβ5.1/5.2$^{+}$Vα2$^{+}$ T cells.

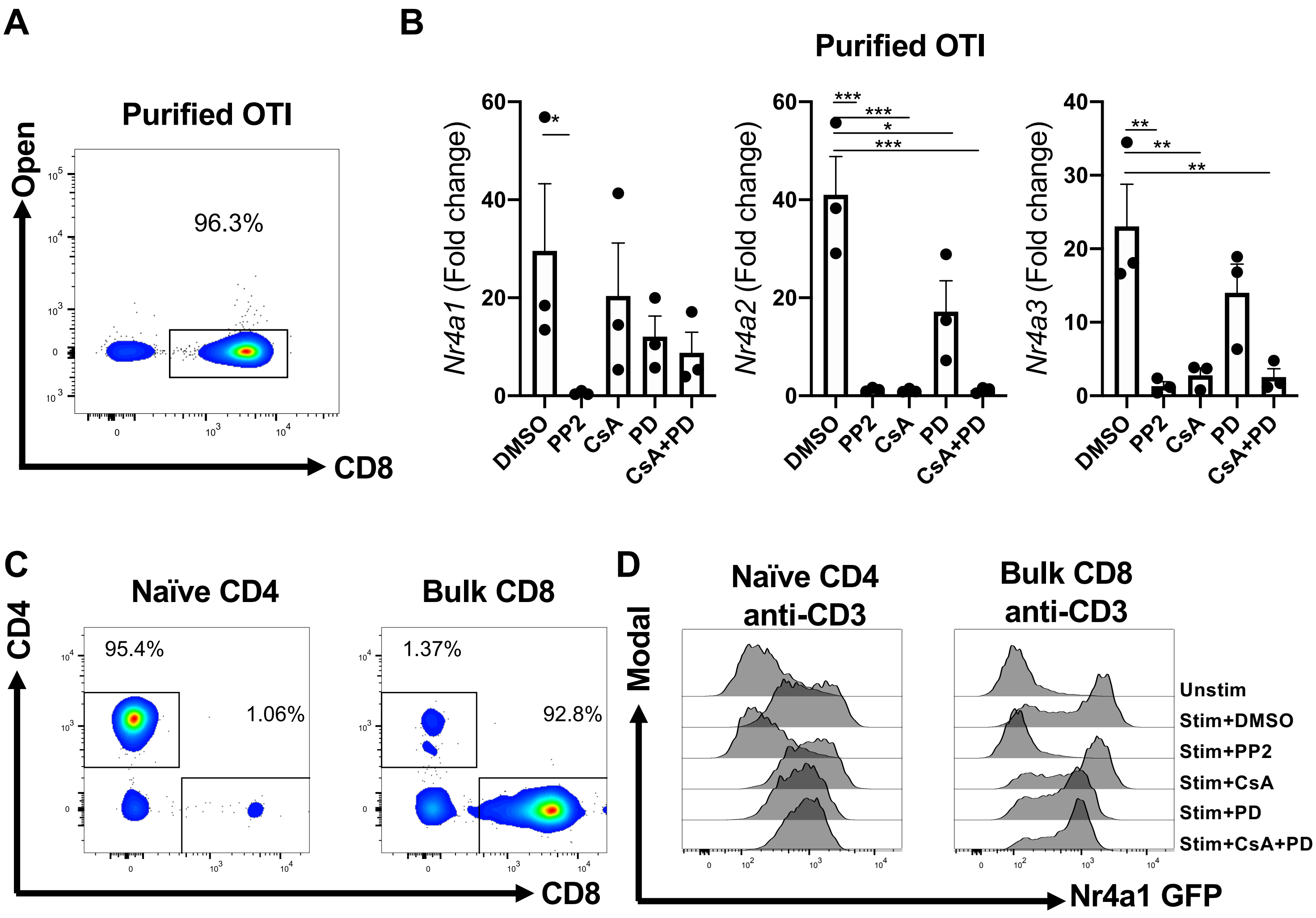


**Supplementary Figure 2: Nr4a receptor expression in purified T cell subsets mirrors responses of bulk splenocyte populations to TCR signalling pathways (related to Figure 2)**

**(A)** $CD8^+$ T cells from OTI Nr4a3-Tocky mice were isolated by immunomagnetic selection, purity of a representative isolation is shown gated on live lymphocytes. (**B**) OTI Nr4a3-Tocky $CD8^+$ T cells were stimulated with 1μM ova peptide for two hours in the presence of DMSO, 10μM PP2, 1μM Cyclosporin A (CsA), 5μM PD0325901 (PD) MEK inhibitor or 1μM Cyclosporin A (CsA)+5μM PD0325901 (PD) MEK inhibitor before RNA extraction. Transcript levels of *Nr4a1, Nr4a2* and *Nr4a3* were measured by qPCR and fold change in expression calculated based on unstimulated controls. n=3, bars represent mean±SEM. Statistical analysis by one-way Anova with Tukey's multiple comparisons test. **(C)** Naïve CD4 (left) or bulk CD8 (right) T cells were isolated by immunomagnetic selection from spleens of Nur77-GFP Nr4a3-Tocky mice, representative purity plots based on CD4 and CD8 expression are shown, hgated on live lymphocyte populations. (**D**) T cell populations were stimulated with 5μg/ml soluble anti-CD3 for six hours in the presence of 0.1% DMSO, 10μM PP2, 1μM Cyclosporin A (CsA), 5μM PD0325901 (PD) MEK inhibitor or 1μM Cyclosporin A (CsA)+5μM PD0325901 (PD) MEK inhibitor. Nr4a1-GFP levels in $CD4^+$ (left) or $CD8^+$ right T cells was then evaluated by flow cytometry. Data in (D) are representative of two independent experiments.

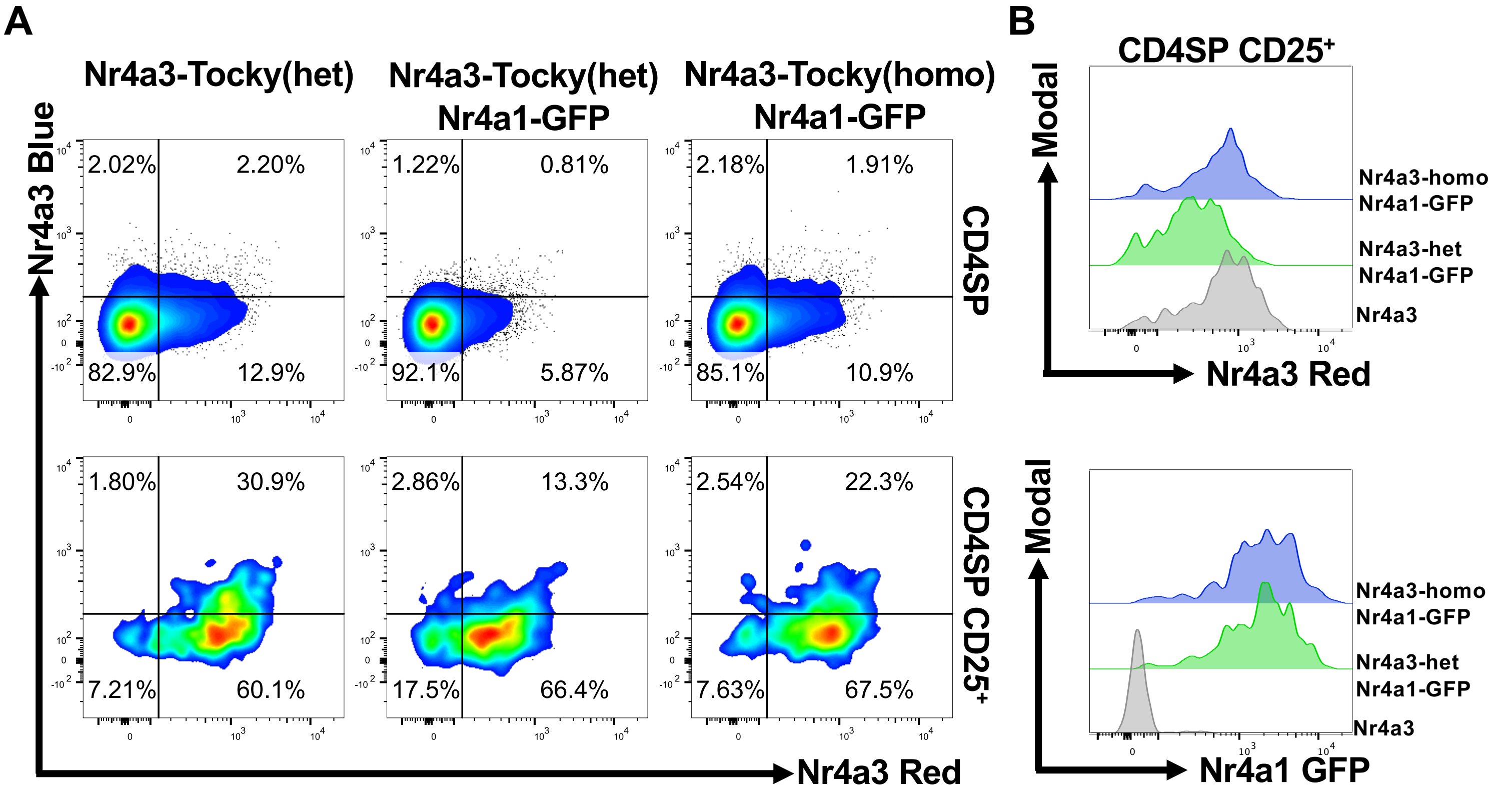


**Supplementary Figure 3: Partial quenching of Nr4a3-Tocky expression when crossed with Nr4a1-GFP line (related to Figure 3)**

(**A**) Nr4a1-GFP Nr4a3-Tocky mice were generated with either heterozygous or homozygous BAC genetic status. CD4SP and CD4SP CD25⁺ T cells within the thymus were compared for Nr4a3-Blue vs. Nr4a3-Red expression. (**B**) Histogram overlays showing Nr4a3-Red (top) or Nr4a1-GFP (bottom) in Nr4a3-heterozygous or Nr4a3-homozygous lines carrying the Nr4a1-GFP transgene.

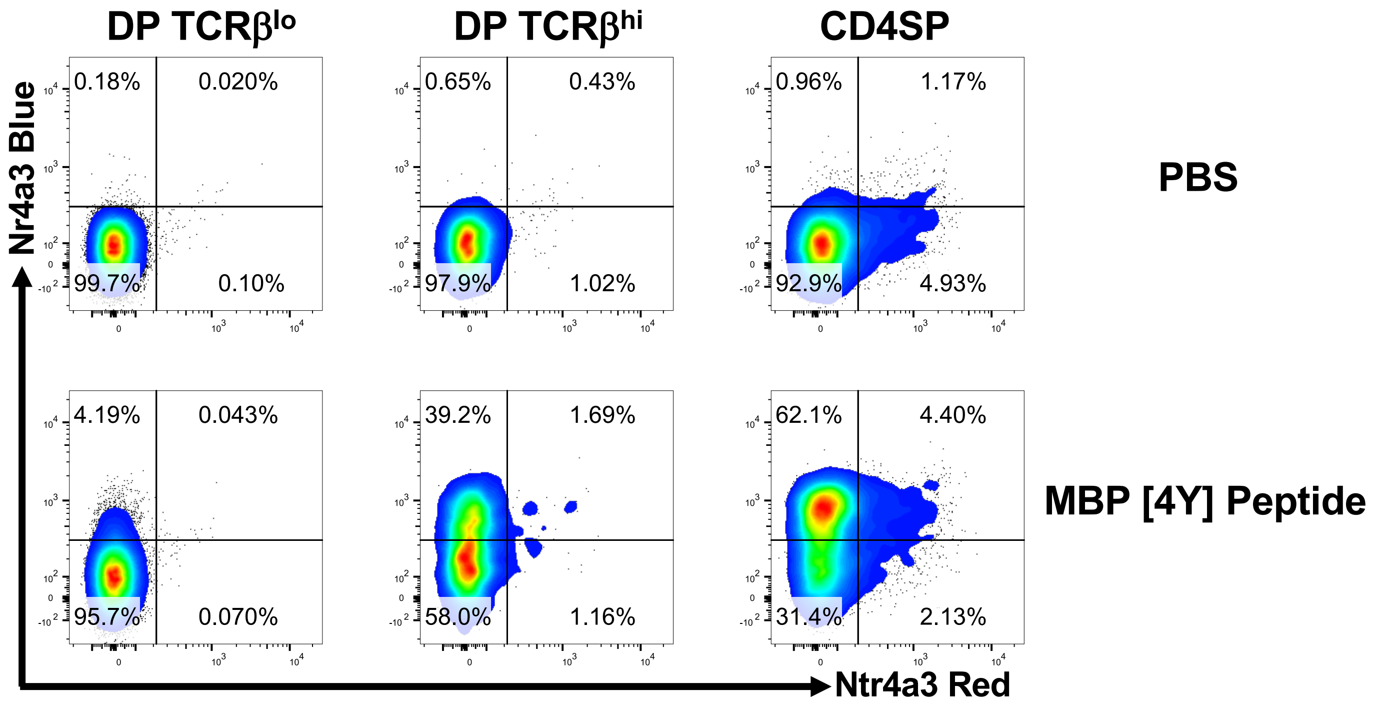


**Supplementary Figure 4: Peptide administration activates Nr4a3 expression in developing thymocytes (related to Figure 3)**

Tg4 Nr4a3-Tocky mice were immunised with 80µg of MBP [4Y] peptide or vehicle control (PBS) and thymus removed 4hrs later and TCRβ$^{lo}$ and TCRβ$^{hi}$ DP and CD4SP subsets analysed for the expression of Nr4a3-Timer Blue vs Timer Red by flow cytometry.

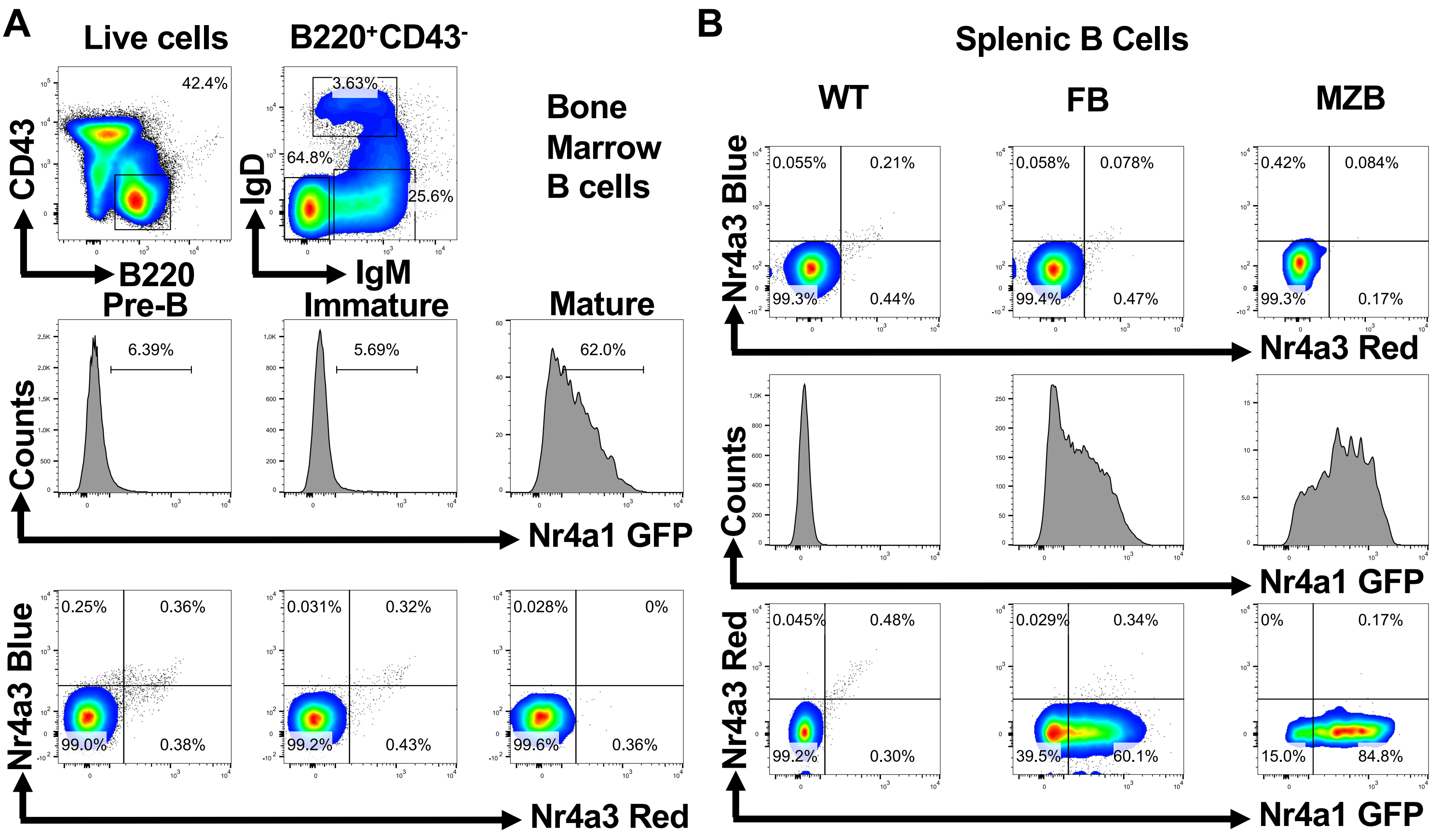


**Supplementary Figure 5: Nr4a1 and Nr4a3 expression patterns during B cell development (related to Figure 3)**

**(A)** Bone marrow from Nr4a1-GFP Nr4a3-Tocky mice was analysed for expression of Nr4a1-GFP and Nr4a3-Timer blue and Nr4a3-Timer Red expression within live B220$^{+}$CD43$^{-}$ B cells. Gating on pre-B cells (B220$^{+}$CD43$^{-}$IgM$^{-}$IgD$^{-}$), immature (B220$^{+}$CD43$^{-}$IgM$^{+}$IgD$^{-}$) and mature B cells (B220$^{+}$CD43$^{-}$IgM$^{+}$IgD$^{+}$) **(B)** Splenic B cell from Nr4a1-GFP Nr4a3-Tocky mice (gated on B220$^{+}$CD19$^{+)}$ were divided into follicular (FB, CD21$^{+}$CD23$^{-}$) and marginal zone (MZB, CD21$^{+}$CD23$^{+}$) subsets and analysed for expression of Nr4a1-GFP and Nr4a3-Timer Blue and Nr4a3-Timer Red expression by flow cytometry. Data are representative of two independent experiments.
